# Quantum coherence, many-body correlations, and non-thermal effects for autonomous thermal machines

**DOI:** 10.1038/s41598-019-39300-4

**Published:** 2019-02-28

**Authors:** C. L. Latune, I. Sinayskiy, F. Petruccione

**Affiliations:** 1 Quantum Research Group, School of Chemistry and Physics, University of KwaZulu-Natal, Durban KwaZulu-Natal, 4001 South Africa; 2 National Institute for Theoretical Physics (NITheP), KwaZulu-Natal, 4001 South Africa; 3 School of Electrical Engineering, KAIST, Daejeon, 34141 Republic of Korea

## Abstract

One of the principal objectives of quantum thermodynamics is to explore quantum effects and their potential beneficial role in thermodynamic tasks like work extraction or refrigeration. So far, even though several papers have already shown that quantum effect could indeed bring quantum advantages, a global and deeper understanding is still lacking. Here, we extend previous models of autonomous machines to include quantum batteries made of arbitrary systems of discrete spectrum. We establish their actual efficiency, which allows us to derive an efficiency upper bound, called maximal achievable efficiency, shown to be always achievable, in contrast with previous upper bounds based only on the Second Law. Such maximal achievable efficiency can be expressed simply in term of the *apparent temperature* of the quantum battery. This important result appears to be a powerful tool to understand how quantum features like coherence but also many-body correlations and non-thermal population distribution can be harnessed to increase the efficiency of thermal machines.

## Introduction

**Figure 1:**
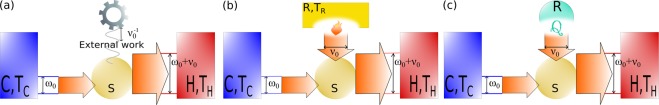
Quantum refrigerators composed of a working medium *S* interacting resonantly at *ω*_0_ with the cold bath *C*. The hot bath *H*, resonant at frequency *ω*_0_ + *ν*_0_, receives quanta of energy *ω*_0_ + *ν*_0_ (*ħ* = 1) from *S* thanks to an energy input *ν*_0_ from (**a**) an external source, or (**b**) a thermal bath *R* at temperature *T*_*R*_, or (**c**) a quantum battery *R* boosted by quantum effects. An engine (promoting energy extraction) is obtained by reverting the energy flows.

Quantum machines aim to attend to technological and experimental needs^1,2^ of nano-scale non-invasive devices capable of cooling or loading energy in single quantum systems (e.g. nano-resonators, cantilevers, atoms). A parallel objective is to explore to which extent genuine quantum effects can assist or enhance the performance of such machines like they do for quantum computation and quantum metrology. Some of the most notorious quantum and non-equilibrium characteristics, quantum coherence and correlations, where shown in^3^ to turn quantum thermodynamics intrinsically different from its classical counterpart. It is therefore essential to investigate what is their impact on quantum machines. Following this objective, several papers on cyclic machines investigate the effect of quantum coherence^4–9^, many-body correlations^9–20^, and other non-thermal characteristics (mainly squeezing)^21–26^ on thermodynamic tasks (refrigeration or work/energy extraction). Similarly, studies investigated the effects of coherence^27^ and correlations^28^ on semi-classical continuous machines (simultaneous and continuous interaction with both the cold and hot baths) driven by external controls (Fig. 1, panel a).

However, such cyclic or semi-classical machines require time-dependent external controls, raising questions regarding the energetic cost of such operations^29^ and doubts about the overall energetic balance of the machines. Moreover, contacts with classical systems (necessary for external controls) make them not well-fitted for non-invasive and local applications. Above all, the study of quantum effects in such machines often requires baths in very unprobable non-thermal states (and very demanding energetically and experimentally to prepare). A more viable alternative to baths is offered by the collisional model. Nevertheless, its requirement of short and repeated interactions together with a high number of reinitializations or preparations of identical systems also represents experimental difficulties.

One promising alternative avoiding the above drawbacks is autonomous machines. Differently from cyclic and semi-classical machines (shown to be equivalent^30^), autonomous machines operate autonomously with no need of external work or controls (which ensures that all energetic and entropic contributions are taken into account). The absence of external controls makes them more suitable for nano-scale and non-invasive operations. Most studies focus on autonomous machines involving three thermal baths, sometimes called absorption refrigerators (Fig. 1, panel b), where one of the bath plays the role of the energy source of the machine^31–49^.

Nevertheless, there is an other type of autonomous machines whose source of energy comes from a quantum battery, namely an ancillary system of finite size (i.e. not a bath) (Fig. 1, panel c). The quantum battery interacts continuously with the machine, offering naturally a platform more adapted and convenient than baths to explore quantum and non-thermal effects. Such machines are more challenging theoretically than semi-classical machines or than absorption refrigerators due to the continuous interaction with the quantum battery which cannot be treated as a bath. Methods used for cyclic machines cannot be applied for these autonomous machines. Instead, one has to establish the dissipative dynamics of the working media together with the quantum battery, which becomes a particularly complex task since their total Hamiltonian is not necessarily diagonalisable (due to their interaction). Such machines received little attention so far. Indeed, it is still unclear whether quantum and non-thermal effects can bring advantages to autonomous thermal machines. Previous studies, pioneered in^50^ and continued in^51–53^ already started to address this problem and provided a general upper bound. However, such upper bound gives little information about the specific role of coherence and correlations. Moreover, its attainability is not certain and not discussed.

Here, we study in details the impact of quantum and non-thermal features on the performance of autonomous thermal machines in a broadly extended framework. After establishing the actual efficiency we provide the maximal achievable efficiency. Interestingly, it can be expressed simply in term of the concept of apparent temperature introduced in^3^. This allows us to investigate straightforwardly the impact on autonomous thermal machines of three non-thermal features: quantum coherence, many-body correlations, and non-thermal population distribution.

## Results

### The model

The aim of a thermal machine is to reverse the natural heat flow between two thermal baths *C* and *H* of different temperature *T*_*C*_ and *T*_*H*_, respectively, or to extract work (or energy) from them. This is achieved by introducing a system *S* interacting with both *C* and *H*. Even though we could realise refrigeration or energy extraction with only one single system, traditionally *S* is used as a connection between the baths and a quantum battery (an ancillary system) which provides or stores energy. We denotes such quantum battery by *R*. The connecting system *S* is often called working medium. For semi-classical or cyclic machines, *R* is not included in the physical description and thus behaves as a classical system. By contrast, in autonomous thermal machines, *R* is included in the physical description and the ensemble *SRCH* is assumed to evolve unitarily through a *time*-*independent* Hamiltonian (ensuring no external source of work). Although no coupling is considered between *R* and the baths, they end up interacting indirectly (through *S*). The global Hamiltonian is *H*_*global*_ = *H*_*S*_ + *H*_*R*_ + *H*_*B*_ + *V*_*SR*_ + *V*_*SB*_, where *H*_*X*_, *X* = *S*, *R*, *B* are free Hamiltonians of the corresponding subsystems and *B* collectively denotes the two baths *C* and *H*. *V*_*SR*_ (*V*_*SB*_) is the coupling Hamiltonian between *S* and *R* (*B*). In the following we regroup the terms *H*_*S*_, *H*_*R*_ and *V*_*SR*_ under the notation ${H}_{SR}\,:\,={H}_{S}+{H}_{R}+{V}_{SR}$.

**Figure 2:**
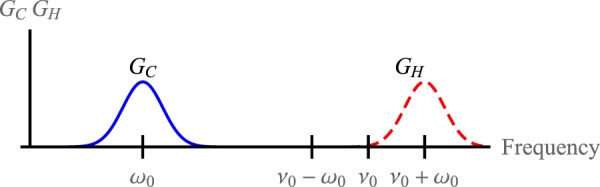
Bath spectral densities *G*_*C*_ and *G*_*H*_ of the baths *C* and *H*, respectively. The cold bath *C* is resonant with the transition energy *ω*_0_ of *S*, whereas the hot bath *H* is resonant with the transition *ω*_0_ + *ν*_0_ of *SR*.

From the point of view of the Second Law, expressed and discussed in Methods (‘Upper bound from the Second Law’), it might seem that one just needs to inject energy and/or entropy in order to realise a thermal machine. However, it is no so simple. One has to design a device whose dynamics actually inverts the natural heat flow. The second law only provides limits on the performance, but gives no clue about how to realise such machines. Our model is an extension of the one introduced in^52,53^. One of the key feature is that *S* and *R* are dispersively coupled through an Hamiltonian of the form *V*_*SR*_ = *gN*_*S*_*A*_*R*_ (*ħ* = 1) where *g* characterises the strength of the coupling, *A*_*R*_ is an observable of *R*, and *N*_*S*_ = *αH*_*S*_ with *α* a positive constant. We justify the use of a dispersive coupling in Methods (‘Why dispersive coupling?’) showing that it seems to be the only universal coupling allowing for refrigeration or energy extraction for any working media. In order to avoid heat leaks and optimise the efficiency the working medium *S* has to be resonant with only one of the two baths (otherwise energy will flow directly through *S* from the hot bath to the cold). The other bath has to be resonant with *SR* in order to get *R* involved in the dynamics. Thermal machines with multiple resonances with the baths are possible although more complex, therefore we focus on the simpler design with only one resonant transition per bath. Then, we assume that *C* is resonant with *S*, denoting by *ω*_0_ the corresponding transition frequency, and *H* is resonant with *SR* at the frequency *ω*_0_ + *ν*_0_ (see Fig. 2), where *ν*_0_ is a transition frequency of *R* (the choice *H* resonant with *ω*_0_ and *C* with *ω*_0_ − *ν*_0_ yields equivalent dynamics).

One should note that *R* can have other transitions. If so, the width of the bath spectral density *G*_*H*_ (*G*_*C*_) of the hot (cold) bath (defined in (34) and (35)) might need to be reduced in order to avoid resonance with these other transitions. Such baths, if not available directly, can be obtained through filtering or coupling to an intermediary two-level system^35,39,42,47,52–54^. Consequently, instead of a harmonic oscillator as quantum battery (as in^51–53^) we can consider a large class of systems, namely arbitrary system of discrete spectrum (such that the resonance conditions can be satisfied through bath engineering when necessary). In principle, the same is valid for *S*. However, in order to simplify the derivation of the main results we restrict *S* to be either a two-level system (as in^52,53^) or a harmonic oscillator, which provides more flexibility and possibilities for experimental realisations.

The coupling with the baths are considered of the following general form *V*_*SB*_ = *A*_*S*_*A*_*B*_, where *A*_*S*_, *A*_*B*_ = *λ*_*H*_*A*_*H*_ + *λ*_*C*_*A*_*C*_ are observables of *S*, *H*, and *C* respectively, and the constant *λ*_*H*_ (*λ*_*C*_) characterises the strength of the coupling between *S* and *H* (*C*). In the following, such constant are included in *A*_*C*_ and *A*_*H*_, respectively.

### Dynamics

Assuming a weak coupling with the baths (*λ*_*H*_ and *λ*_*C*_ much smaller than the inverse of the bath correlation times) we use the Born-Markov approximation and the formalism of^55^ to derive corrections to the local approach^56^ of the master equation of *SR*. The exact global approach^57,58^ is intractable in general. The main steps of the derivation are described in Methods ‘Expression of the baths’ dissipative operators’. The effects of *V*_*SR*_ are taken into account up to second order in *g*/*ν*_0_ for weak coupling $g\ll {\nu }_{0}$ and $g\ll {\lambda }_{C}$, *λ*_*H*_. Under such conditions *R* evolves slowly through the (indirect) influence of the bath *H*, at a timescale ${\tau }_{R}\,:\,={({G}_{C}({\omega }_{0})\frac{{g}^{2}}{|\nu {|}^{2}})}^{-1}$. *τ*_*R*_ is much larger than the timescale ${\tau }_{es}\,:\,={[{G}_{C}({\omega }_{0})]}^{-1}$ under which *S* is brought to a quasi steady state (thermal state at temperature *T*_*C*_ with corrections of order *g*/*ν*_0_) due to its resonant interaction with *C*. Moreover, defining the internal energy of the subsystem *X* = *R*, *S*, or *RS* as ${E}_{X}\,:\,={\langle {H}_{X}\rangle }_{{\rho }_{SR}}$ (using the notation ${\langle {\mathscr{O}}\rangle }_{\rho }$ to denote ${\rm{Tr}}\rho {\mathscr{O}}$ the expectation value of the operator ${\mathscr{O}}$ in the state *ρ*), one can show that ${\dot{E}}_{S}={\mathscr{O}}({g}^{3}/{\nu }_{0}^{3})$ for times much larger than *τ*_*es*_. It means that *S* can be regarded as “energetically in steady state” so that the energy (in form of quanta *ν*_0_) slowly provided by *R* is transferred to *H* together with energy taken from *C* (in form of quanta *ω*_0_) in order to satisfy the resonance condition of *H* (*ω*_0_ + *ν*_0_). This leads to the refrigeration of *C* and happens only if the condition (5) is fulfilled. Eventually, after long times (much larger than *τ*_*R*_) *R* reaches a steady state and the machine stops working. This is the price to pay for using finite size quantum batteries. In the remainder of the paper we consider the above regime of $t\gg {\tau }_{es}$.

The heat flow from the bath *j* = *H*, *C* to the ensemble *SR* is defined as^59^

1 $${\dot{Q}}_{SR/j}\,:\,={{\rm{Tr}}}_{SR}{ {\mathcal L} }_{j}{\rho }_{SR}^{I}{H}_{SR},$$

where ${\rho }_{SR}^{I}$ denotes the density matrix in the interaction picture with respect to *H*_*SR*_, and the dissipator ${ {\mathcal L} }_{j}$ corresponds to the action of the bath *j* = *H*, *C* on *SR*. According to the above definitions the following equality holds (First Law) ${\dot{E}}_{SR}={\dot{Q}}_{SR/H}+{\dot{Q}}_{SR/C}$. For times much larger than *τ*_*es*_, the heat flow leaving the cold bath, obtained by introducing the expression of ${ {\mathcal L} }_{C}$ in (1), is (see details in Methods and Supplementary Information)

2 $$\begin{array}{lll}{\dot{Q}}_{SR/C} & \propto  & (\frac{{g}^{2}}{{\nu }_{0}^{2}})\,{\omega }_{0}\,({e}^{\tfrac{-{\omega }_{0}}{{T}_{C}}}{\langle {A}_{R}^{\dagger }({\nu }_{0}){A}_{R}({\nu }_{0})\rangle }_{{\rho }_{R}^{I}}-\,{e}^{\tfrac{-({\omega }_{0}+{\nu }_{0})}{{T}_{H}}}{\langle {A}_{R}({\nu }_{0}){A}_{R}^{\dagger }({\nu }_{0})\rangle }_{{\rho }_{R}^{I}})+\,{\mathscr{O}}(\frac{{g}^{3}}{{\nu }_{0}^{3}}).\end{array}$$

The above expression is a generalisation of the one obtained in^52,53,60^ for harmonic oscillators (the Boltzmann constant is set equal to 1). *A*_*R*_(*ν*_0_) and ${A}_{R}^{\dagger }({\nu }_{0})$ are the eigenoperators for the transition energy *ν*_0_, defined by ${A}_{R}({\nu }_{0})={\sum }_{\varepsilon ^{\prime} -\varepsilon ={\nu }_{0}}\,{\rm{\Pi }}(\varepsilon ){A}_{R}{\rm{\Pi }}(\varepsilon ^{\prime} )$ (and the hermitian conjugate for ${A}_{R}^{\dagger }({\nu }_{0})$), with Π(*ε*) being the projector onto the eigenspace of *H*_*R*_ associated to the eigenenergy *ε*. Moreover, the following relations hold for $t\gg {\tau }_{es}$,

3 $$\frac{{\dot{Q}}_{SR/C}}{{\omega }_{0}}=-\,\frac{{\dot{Q}}_{SR/H}}{{\omega }_{0}+{\nu }_{0}}+{\mathscr{O}}(\frac{{g}^{3}}{{\nu }_{0}^{3}})=-\,\frac{{\dot{E}}_{R}}{{\nu }_{0}}+{\mathscr{O}}(\frac{{g}^{3}}{{\nu }_{0}^{3}}).$$

### Actual (universal) efficiency

We focus firstly on refrigeration. The energy extraction regime will be treated in a second time. The efficiency refrigeration *η*_*r*_ is defined as the ratio of the heat extracted from *C*, accounted by ${\dot{Q}}_{SR/C}$, by the energy invested (and provided by *R*), accounted by −${\dot{E}}_{R}$, ${\eta }_{r}\,:=\frac{{\dot{Q}}_{SR/C}}{-{\dot{E}}_{R}}$. From the above relation (3) we deduce that

4 $${\eta }_{r}=\frac{{\omega }_{0}}{{\nu }_{0}}+{\mathscr{O}}({g}^{3}/{\nu }_{0}^{3}).$$

Remarkably, the efficiency is constant and independent from the initial state of *R*. This is contrasting with the intuition created by the Second Law and its associated upper bound (19). Equation (4) extends to quantum batteries of discrete spectrum in any state the result already known for thermal two-level systems or thermal baths^38,46,54^. However, one should note that even though the actual efficiency does not depend on the initial state of *R*, the power of the machine does depend on it, but also the operating regime (refrigeration or energy extraction). Alternatively, for a given state of *R*, one can ask what is the maximum achievable efficiency (adjusting parameters of the setup like the resonance *ω*_0_). This is the object of the next paragraph.

### Maximal achievable efficiency

From (4) one can see that in order to increase the efficiency one only needs to increase *ω*_0_. However, if *ω*_0_ is too large, the refrigerator may stop refrigerating. Then, the value of *ω*_0_ has to be subjected to the constraint that (2) should remain positive, ${\dot{Q}}_{SR/C}\ge 0$. According to (3) this happens simultaneously with ${\dot{E}}_{R}\le 0$, meaning that *R* is powering the machine. This leads to the following necessary and sufficient condition for refrigeration,

5 $${\omega }_{0}\le {\nu }_{0}\frac{{T}_{C}}{{T}_{H}-{T}_{C}}(1-\frac{{T}_{H}}{{{\mathscr{T}}}_{R}}),$$

where ${{\mathscr{T}}}_{R}$ is the *apparent temperature* of *R*, defined as

6 $${{\mathscr{T}}}_{R}\,:\,={\nu }_{0}\,{(\mathrm{ln}\frac{{\langle {A}_{R}({\nu }_{0}){A}_{R}^{\dagger }({\nu }_{0})\rangle }_{{\rho }_{R}^{I}}}{{\langle {A}_{R}^{\dagger }({\nu }_{0}){A}_{R}({\nu }_{0})\rangle }_{{\rho }_{R}^{I}}})}^{-1},$$

and introduced in^3^. The above Eq. (5) leads straightforwardly to the *maximal achievable efficiency η*_ac_ (achieved at zero power, as usual),

7 $${\eta }_{r}=\frac{{\omega }_{0}}{{\nu }_{0}}+{\mathscr{O}}(\frac{{g}^{3}}{{\omega }_{0}^{3}})\le {\eta }_{{\rm{a}}{\rm{c}}}\,:=\frac{{T}_{C}}{{T}_{H}-{T}_{C}}\,(1-\frac{{T}_{H}}{{{\mathscr{T}}}_{R}}).$$

The apparent temperature was shown to determine the heat flows between out-of-equilibrium quantum systems and general reservoir (bath or collisional model)^3^, appearing as a quantifier of a system’s tendency to cede packets of quantised energy. For thermal states it coincides with the usual temperature (appearing in the Boltzmann distribution). It is therefore remarkable that the maximal achievable efficiency can be expressed in term of the apparent temperature of *R*. This brings a physically meaningful upper bound: when *R* is in a thermal state, the achievable upper bound is given by the usual Carnot bound, and when *R* is in a non-thermal state, the achievable maximal efficiency is simply given by substituting the temperature by the apparent temperature. Furthermore, this re-enforces and extends the relevance of the concept of apparent temperature beyond its original framework.

Finally, this result is important for two more reasons. First, it provides an *achievable* maximal efficiency, which is not provided by previous upper bounds derived from the Second Law. The main reason is that the upper bound (19) deduced from the second law is expressed in term of $\dot{S}({\rho }_{R})$, the entropy change rate of *R*. On the other hand, this upper bound is supposed to be reached when the entropy production rate ${\dot{{\rm{\Sigma }}}}_{R}$ (always positive) is equal to zero (sometimes also called the reversibility condition^61^). However, when ${\dot{{\rm{\Sigma }}}}_{R}$ is equal to zero, $\dot{S}({\rho }_{R})$ is smaller. In other words, when the upper bound is supposed to be saturated the value of $\dot{S}({\rho }_{R})$ has changed, bringing doubts whether the upper bound (19) can ever be reached. Moreover, it is not guaranteed that one can ever engineer a machine such that ${\dot{{\rm{\Sigma }}}}_{R}=0$. Such problematics regarding upper bounds were recently discussed in^61^ (for cyclic machines). From an other perspective, the upper bound (19) gives the misleading idea that the efficiency can be increased by increasing the entropy change rate $\dot{S}({\rho }_{R})$. This is not true in general. The actual efficiency can indeed be expressed as ${\eta }_{r}=\frac{{T}_{C}}{{T}_{H}-{T}_{C}}(1+{T}_{H}\frac{{\dot{S}}_{{\rm{fl}}}({\rho }_{R})}{-\,{\dot{E}}_{R}})$, obtained by substituting in (19) the entropy change rate $\dot{S}({\rho }_{R})$ by the flow of entropy ${\dot{S}}_{{\rm{fl}}}({\rho }_{R})\,:\,=\dot{S}({\rho }_{R})-{\dot{{\rm{\Sigma }}}}_{R}$. Then, increasing $\dot{S}({\rho }_{R})$ is not a guarantee of increase of ${\dot{S}}_{{\rm{fl}}}({\rho }_{R})$ and therefore of the efficiency. It has been noted for instance that coherences between non-degenerate levels increase the entropy production but do not affect the flow of entropy^62^.

Secondly, using the framework introduced in^3^ the result (7) provides crucial information on how quantum and non-equilibrium effects can be harnessed to boost quantum thermal machines. This is detailed in the following paragraphs.

### Recovering known results

We first show that the maximum achievable efficiency (7) provides the usual results for the known situations. As already mentioned above, when *R* is in a thermal state, ${{\mathscr{T}}}_{R}$ coincides with the usual temperature (appearing in the Boltzmann distribution)^3^ and the usual Carnot bound^34,35,37–39,45^ is recovered from (7). In the limit of classical batteries, the ladder operators commute, $[{A}_{R}({\nu }_{0}),{A}_{R}^{\dagger }({\nu }_{0})]=0$, and then the associated apparent temperature is ${{\mathscr{T}}}_{R}=+\,\infty$, implying that *R* behaves as a pure work reservoir. We recover the well-known observation that classical work reservoirs correspond to infinite-temperature thermal baths^63^. Finally, if *R* is a harmonic oscillator in a squeezed thermal state, its apparent temperature can be expressed in terms of the temperature of the thermal excitation *T*_*R*_ and the squeezing factor *r* as (see Supplementary Information), ${{\mathscr{T}}}_{R}={\nu }_{0}\,{[{\rm{l}}{\rm{n}}{\textstyle \tfrac{{\tanh }^{2}r+{e}^{{\nu }_{0}/{T}_{R}}}{{{e}^{{\nu }_{0}/{T}_{R}}\tanh }^{2}r+1}}]}^{-1}$, so that when substituting in (7) the maximal achievable efficiency is the analogue of the upper bound derived in^26,38^ for machines powered by squeezed thermal baths.

### Effects of coherence

**Figure 3:**
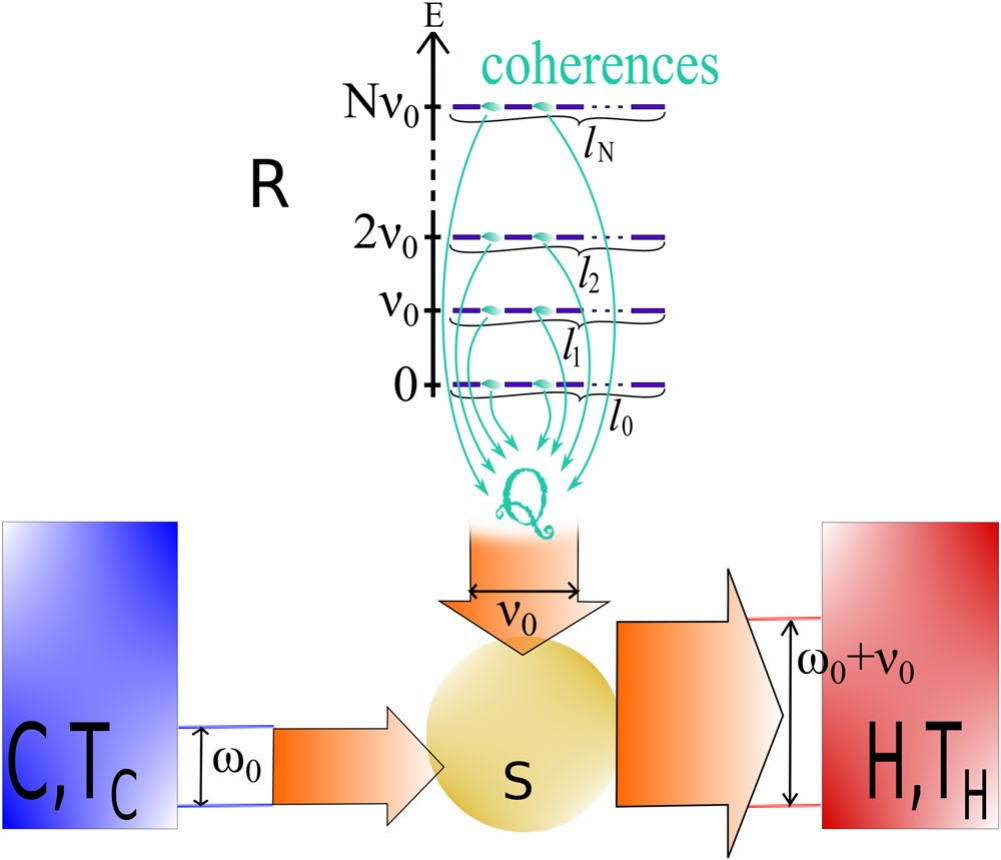
Example of a degenerated-level system *R* used to power an autonomous refrigerator. The energy levels |*n*, *k*〉, *n* ∈ [0; *N*], *k* ∈ [1; *l*_*n*_], are represented by purple dashes, with the vertical axis representing the energy of the levels and the horizontal axis the degeneracy. The presence of coherence between degenerated levels (represented by a green link) affect dramatically the apparent temperature. Higher efficiencies are achievable if and only if the coherences satisfy the condition (11).

We now combine the framework and results from^3^ to (7). Only levels taking part in transitions of energy *ν*_0_ contribute to the heat flows (due to the resonance condition). Then, if *R* has (*N* + 1) energy levels involved in such transitions, we denote them by |*n*, *k*〉 with *n* ∈ [0; *N*] and *k* is the degeneracy index running from 1 to *l*_*n*_ ≥ 1, *l*_*n*_ being the number of degeneracy of the level *n* (see Fig. 3). In other words, *H*_*R*_|*n*, *k*〉 = *nν*_0_|*n*, *k*〉 with *n* ∈ [0; *N*] and *k* ∈ [1; *l*_*n*_]. The ladder operator *A*_*R*_(*ν*_0_) can be expressed as (using the expression mentioned after (2)),

8 $${A}_{R}({\nu }_{0})=\sum _{n=1}^{N}\,\sum _{k=1}^{{l}_{n-1}}\,\sum _{k^{\prime} =1}^{{l}_{n}}\,{\alpha }_{n-1,n,k,k^{\prime} }|n-1,k\rangle \langle n,k^{\prime} |,$$

with ${\alpha }_{n-1,n,k,k^{\prime} }\,:\,=\langle n-1,k|{A}_{R}|n,k^{\prime} \rangle$. We assume that all transitions amplitudes *α*_*n*−1,*n*,*k*,*k*′_ are equal as it does not change the nature of the results and simplify the expressions. The situation where *R* is an infinite-level system (harmonic oscillator) is treated in the following. From the definition (6) we find for the apparent temperature^3^

9 $${{\mathscr{T}}}_{R}={\nu }_{0}\,{(\mathrm{ln}\frac{{\sum }_{n=1}^{N}{l}_{n}({\rho }_{n-1}+{c}_{n-1})}{{\sum }_{n=1}^{N}{l}_{n-1}({\rho }_{n}+{c}_{n})})}^{-1},$$

where ${\rho }_{n}\,:\,={\sum }_{k=1}^{{l}_{n}}\,\langle n,k|{\rho }_{R}|n,k\rangle$ is the sum of the populations of the degenerate levels of energy *nω*, and ${c}_{n}\,:\,={\sum }_{k\ne k^{\prime} \in [1,{l}_{n}]}\,\langle n,k|{\rho }_{R}|n,k^{\prime} \rangle$ is the sum of the coherences between these same degenerate levels. The corresponding maximal achievable efficiency can be re-written as

10 $${\eta }_{{\rm{ac}}}=\frac{{T}_{C}}{{T}_{H}-{T}_{C}}\,[1-\frac{{T}_{H}}{{{\mathscr{T}}}_{0}}-\frac{{T}_{H}}{{\nu }_{0}}\,\mathrm{ln}\,\frac{1+{{\mathscr{C}}}^{-}/{\rho }^{-}}{1+{{\mathscr{C}}}^{+}/{\rho }^{+}}],$$

where ${{\mathscr{T}}}_{0}\,:\,={\nu }_{0}{(\mathrm{ln}{\rho }^{-}/{\rho }^{+})}^{-1}$ is the apparent temperature of *R* without coherence (that is all *c*_*n*_ equal to 0), and we defined ${{\mathscr{C}}}^{+}\,:\,={\sum }_{n=1}^{N}\,{l}_{n-1}{c}_{n}$, ${\rho }^{+}\,:\,={\sum }_{n=1}^{N}\,{l}_{n-1}{\rho }_{n}$, ${{\mathscr{C}}}^{-}\,:\,={\sum }_{n=1}^{N}\,{l}_{n}{c}_{n-1}$, and ${\rho }^{-}\,:\,={\sum }_{n=1}^{N}\,{l}_{n}{\rho }_{n-1}$.

The result (10) is important as it provides how coherence affects the efficiency. In particular, it is important to note that coherences between levels of different energy do not affect the apparent temperature and therefore do not confer possibilities of efficiency increase. The core mechanism relies on the fact that heat exchanges are controlled by the quantities ${\langle {A}_{R}({\nu }_{0}){A}^{\dagger }({\nu }_{0})\rangle }_{{\rho }_{R}}$ and ${\langle {A}_{R}^{\dagger }({\nu }_{0})A({\nu }_{0})\rangle }_{{\rho }_{R}}$ determined by the populations but also by coherences between degenerated states of *R* which ends up affecting the apparent temperature^3^ and the achievable efficiency (see Fig. 3). The overall balance, which is not always beneficial for the efficiency, is reflected in (10). Coherences increase the maximal achievable efficiency if and only if

11 $${{\mathscr{C}}}^{+}\ge {{\mathscr{C}}}^{-}{e}^{-{\nu }_{0}/{{\mathscr{T}}}_{0}}.$$

Moreover, ${{\mathscr{C}}}^{\pm }$ can take value close to *ρ*^±^ (with the restriction of positivity and ${{\mathscr{C}}}^{+}+{\rho }^{+}\ge 0$ and ${{\mathscr{C}}}^{-}+{\rho }^{-}\ge 0$), which can generate great increase of achievable efficiency.

As an example we mention the phaseonium^4,5^, which is a three-level system with exited state |*a*〉 and two (quasi-)degenerated ground states |*b*〉 and |*c*〉 (sometimes referred as the Λ-configuration). For such system, ${{\mathscr{C}}}^{+}=0$, ${{\mathscr{C}}}^{-}={c}_{0}=\langle b|{\rho }_{R}|c\rangle +\langle c|{\rho }_{R}|b\rangle$, and *ρ*^−^ = *ρ*_0_ = 〈*b*|*ρ*_*R*_|*b*〉 + 〈*c*|*ρ*_*R*_|*c*〉, so that the maximal achievable efficiency would be ${\eta }_{{\rm{ac}}}=\tfrac{{T}_{C}}{{T}_{H}-{T}_{C}}\,[1-\tfrac{{{\mathscr{T}}}_{H}}{{{\mathscr{T}}}_{0}}-\tfrac{{T}_{H}}{{\nu }_{0}}\,\mathrm{ln}(1+{c}_{0}/{\rho }_{0})]$. Then, one finds that the coherence in the phaseonium increases the maximal achievable efficiency if and only if *c*_0_ < 0, yielding large increases when *c*_0_ takes values close to −*ρ*_0_.

Conversely, if the system used is a three-level system in the *V*-configuration (|*a*〉 is the ground state, |*b*〉 and |*c*〉 are the degenerated excited states), the maximal achievable efficiency takes the form, $${\eta }_{{\rm{ac}}}=\tfrac{{T}_{C}}{{T}_{H}-{T}_{C}}$$$$[1-\tfrac{{T}_{H}}{{{\mathscr{T}}}_{0}}+\tfrac{{T}_{H}}{{\nu }_{0}}\,\mathrm{ln}(1+{c}_{1}/{\rho }_{1})]$$, with *c*_1_ = 〈*b*|*ρ*_*R*_|*c*〉 + 〈*c*|*ρ*_*R*_|*b*〉 and *ρ*_1_ = 〈*b*|*ρ*_*R*_|*b*〉 + 〈*c*|*ρ*_*R*_|*c*〉. The conclusion is opposite to the previous one: coherence increases the maximal achievable efficiency if and only if *c*_1_ > 0.

### Effects of correlations

**Figure 4:**
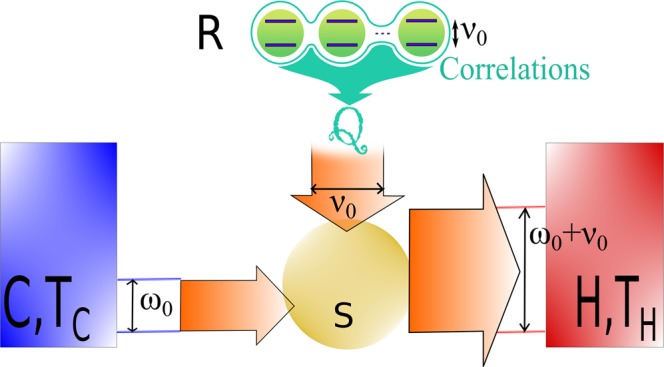
Example of a many-body system (here, an ensemble of two-level systems) powering an autonomous refrigerator. The presence of correlations affect dramatically the apparent temperature and higher efficiencies are achievable if and only if the condition (14) is satisfied.

In this paragraph we assume that *R* is an ensemble of *N* non-interacting subsystems (not necessarily identical neither with finite number of levels) of *same* transition energy *ν*_0_ (Fig. 4). We require the following important condition that all the *N* subsystems appear indistinguishable to *S* (which usually requires confinement in a volume smaller than typical length scales of *S*). Upon the above conditions the *N* sub-systems interact *collectively* with *S* and the ladder operator *A*_*R*_(*ν*_0_) is a *collective* ladder operator, ${A}_{R}({\nu }_{0})={\sum }_{i=1}^{N}\,{A}_{i}({\nu }_{0})$, where *A*_*i*_(*ν*_0_) is the ladder operator of the subsystem *i* (with the same properties as above). Then, applying definition (6), the apparent temperature of the ensemble *R* is,

12 $${{\mathscr{T}}}_{R}={\nu }_{0}\,{(\mathrm{ln}\frac{{\sum }_{i=1}^{m}{\langle {A}_{i}({\nu }_{0}){A}_{i}^{\dagger }({\nu }_{0})\rangle }_{{\rho }_{R}}+c}{{\sum }_{i=1}^{m}{\langle {A}_{i}^{\dagger }({\nu }_{0}){A}_{i}({\nu }_{0})\rangle }_{{\rho }_{R}}+c})}^{-1},$$

where *c* is the sum of the correlations (and product of local coherences, see^3^), $c\,:\,={\sum }_{i\ne j=1}^{m}\,{\langle {A}_{i}({\nu }_{0}){A}_{j}^{\dagger }({\nu }_{0})\rangle }_{{\rho }_{R}}$ = ${\sum }_{i\ne j=1}^{m}\,{\langle {A}_{i}^{\dagger }({\nu }_{0}){A}_{j}({\nu }_{0})\rangle }_{{\rho }_{R}}$. As previously with coherence, *correlations act as populations*, and their impact on the apparent temperature can be significant, enabling high efficiency increases. The maximal achievable efficiency is

13 $${\eta }_{{\rm{ac}}}=\frac{{T}_{C}}{{T}_{H}-{T}_{C}}\,[1-\frac{{T}_{H}}{{{\mathscr{T}}}_{0}}-\frac{{T}_{H}}{{\nu }_{0}}\,\mathrm{ln}\,\frac{1+c/{n}^{-}}{1+c/{n}^{+}}],$$

where ${{\mathscr{T}}}_{0}\,:\,={\nu }_{0}{(\mathrm{ln}{n}^{-}/{n}^{+})}^{-1}$ is the apparent temperature in the absence of correlation, and we defined ${n}^{-}\,:\,={\sum }_{i=1}^{m}\,{\langle {A}_{i}({\nu }_{0}){A}_{i}^{\dagger }({\nu }_{0})\rangle }_{{\rho }_{R}}$, and ${n}^{+}\,:\,={\sum }_{i=1}^{m}\,{\langle {A}_{i}^{\dagger }({\nu }_{0}){A}_{i}({\nu }_{0})\rangle }_{{\rho }_{R}}$.

The same comments made in the previous paragraph on the role of coherence are valid here as well. The achievable efficiency is increased if and only if

14 $$c({e}^{{\nu }_{0}/{{\mathscr{T}}}_{0}}-1)\ge 0,$$

or in other words if and only if *c* ≥ 0 when ${{\mathscr{T}}}_{0}\ge 0$ (and the opposite when ${{\mathscr{T}}}_{0}\le 0$).

As illustrative example we mention *N* two-level systems in Dicke states (Fig. 4), $$|N,{n}_{e}\rangle \,:\,=$$$$\sqrt{\frac{{n}_{e}!}{N!{n}_{g}!}}{({\sum }_{i=1}^{N}{\sigma }_{i}^{-})}^{{n}_{g}}{\otimes }_{i=1}^{N}|e{\rangle }_{i}$$^64,65^, where *n*_*e*_ represents the number of delocalised excitations and $${n}_{g}\,:\,=N-{n}_{e}$$ is the number of ground states. The corresponding maximal achievable efficiency is $${\eta }_{{\rm{ac}}}=\frac{{T}_{C}}{{T}_{H}-{T}_{C}}[1-\frac{{T}_{H}}{{{\mathscr{T}}}_{0}}-\frac{{T}_{H}}{{\nu }_{0}}\,\mathrm{ln}\,\frac{1+{n}_{e}}{1+{n}_{g}}]$$, where $${{\mathscr{T}}}_{0}\,:\,={\nu }_{0}{(\mathrm{ln}\frac{{n}_{g}}{{n}_{e}})}^{-1}$$. It appears then that the correlations present in the Dicke state increase the maximal achievable efficiency only for non-inverted states (*n*_*g*_ ≥ *n*_*e*_), equivalent to $${{\mathscr{T}}}_{0}\ge 0$$. As a comparison, we consider harmonic oscillators in a collective excitation state (analogue to Dicke states), and we obtain $${\eta }_{{\rm{ac}}}=\frac{{T}_{C}}{{T}_{H}-{T}_{C}}[1-\frac{{T}_{H}}{{{\mathscr{T}}}_{0}}-\frac{{T}_{H}}{{\nu }_{0}}\,\mathrm{ln}\,\frac{{n}_{e}}{{n}_{e}+N}]$$, where as previously *n*_*e*_ stands for the number of collective excitations and *N* is the number of harmonic oscillators. Interestingly, although the correlations increase with the number of excitations *n*_*e*_, the impact on the apparent temperature shrinks away with *n*_*e*_ (but the power is increased).

### Effect of non-thermal population distribution

**Figure 5:**
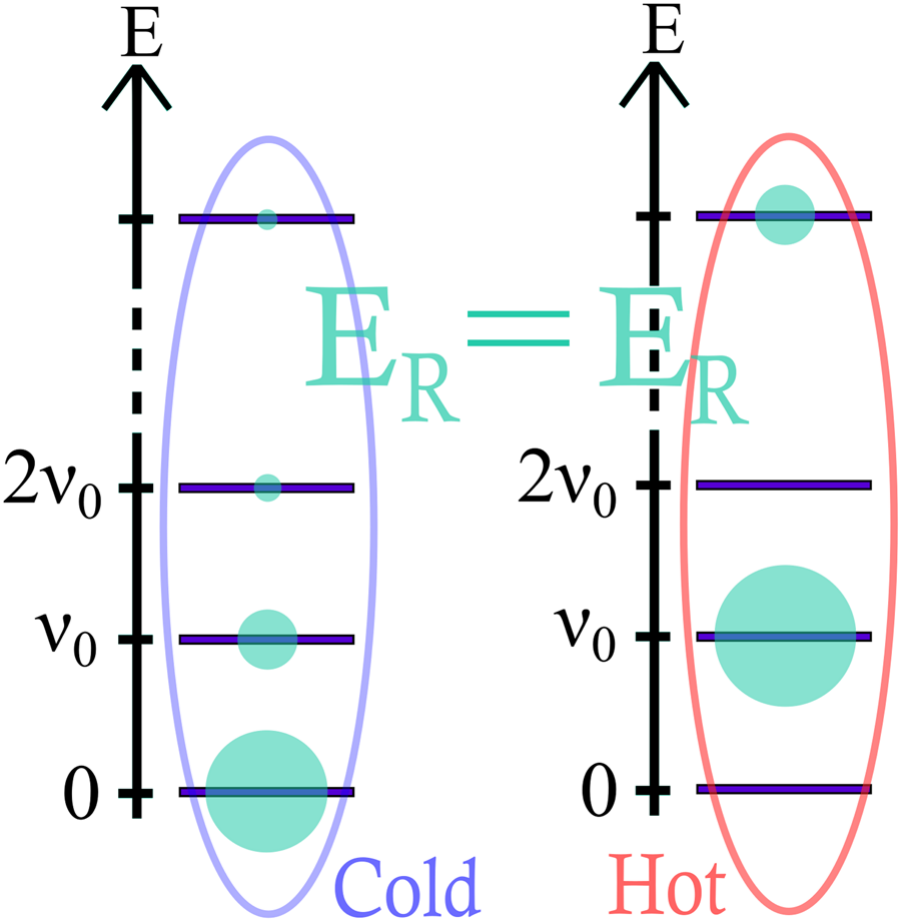
Example of non-thermal population distribution (right-hand side) in a (N + 1)-level system compared to a thermal distribution (left-hand side). Although both states have the same average energy *E*_*R*_, the non-thermal distribution is “hotter” than the thermal one.

No benefit from the above results can be obtained if *R* is a non-degenerated single system. However, alternative non-thermal features can be found to increase the apparent temperature and therefore the maximal achievable efficiency of the machine. For thermal states, temperature and mean energy are in one-to-one correspondence. By contrast, as long as *H*_*R*_ is not proportional to ${A}_{R}^{\dagger }({\nu }_{0}){A}_{R}({\nu }_{0})$, the apparent temperature is not determined by the average energy *E*_*R*_. This implies that, differently from thermal states, non-thermal states of same energy *E*_*R*_ can have *different apparent temperature*. Then, non-thermal population distributions can appear hotter (lower inverse apparent temperature) than thermal states of same energy *E*_*R*_, providing therefore a higher efficiency (see Fig. 5). For a system of *N* + 1 levels (of transition energy *ν*_0_), the apparent temperature can be re-written as ${{\mathscr{T}}}_{R}={\nu }_{0}{(\mathrm{ln}\frac{1-{\rho }_{N}}{1-{\rho }_{0}})}^{-1}$, where *ρ*_*N*_ and *ρ*_0_ denote the populations in the most excited state and in the ground state, respectively, yielding a maximal achievable efficiency of

15 $${\eta }_{{\rm{ac}}}=\frac{{T}_{C}}{{T}_{H}-{T}_{C}}\,[1-\frac{{T}_{H}}{{\nu }_{0}}\,\mathrm{ln}\,\frac{1-{\rho }_{N}}{1-{\rho }_{0}}].$$

As an example, for a 3-level system, one can exhibit non-thermal states with values of ${{\mathscr{T}}}_{R}/{\nu }_{0}$ up to 50% higher than the temperature of thermal states of same average energy *E*_*R*_. For *N* ≥ 4 this is even more drastic since one can find non-thermal states with ${{\mathscr{T}}}_{R}$ equal to infinity or even negative while their thermal state counterparts of same energy have positive and finite temperatures (see Supplementary Information).

We look now at the most common infinite-level system, the harmonic oscillator (of frequency *ν*_0_). The eigenoperators are the usual operator of creation and annihilation, *a* = *A*_*R*_(*ν*_0_) and ${a}^{\dagger }={A}_{R}^{\dagger }({\nu }_{0})$. Then, *H*_*R*_ is proportional to ${A}_{R}^{\dagger }({\nu }_{0}){A}_{R}({\nu }_{0})={a}^{\dagger }a$, which implies that the apparent temperature and the maximal achievable efficiency are completely determined by *E*_*R*_. In other words, there is no special effects from non-thermal states. This points out that no matter under which form the energy is stored in *R*, it powers the refrigerator with the same efficiency. In particular, the same increase of efficiency achieved by squeezing can be achieved with temperature increase by investing *the same amount of energy*: squeezed states are as efficient as thermal states.

### Energy Extraction

The above considerations can be straightforwardly extended to energy extraction regime. Such machines operate with opposite heat flows as compared with the above refrigerators, and thus the extracting conditions are found to be opposite to the refrigeration condition (5),

16 $${\omega }_{0}\ge {\nu }_{0}\frac{{T}_{C}}{{T}_{H}-{T}_{C}}\,(1-\frac{{T}_{H}}{{{\mathscr{T}}}_{R}}).$$

The efficiency *η*_*e*_ of the energy extraction process is naturally defined by the amount of extracted energy, accounted by ${\dot{E}}_{R}$, divided by the energy invested, accounted by ${\dot{Q}}_{SR/H}$, ${\eta }_{e}\,:\,={\dot{E}}_{R}/{\dot{Q}}_{SR/H}$. From (3) one obtains the following expression for the efficiency, ${\eta }_{e}={\nu }_{0}/({\omega }_{0}+{\nu }_{0})+{\mathscr{O}}({g}^{3}/{\nu }_{0}^{3})$, which, as for the refrigeration regime, is constant. The corresponding upper bound follows directly from (16),

17 $${\eta }_{e}\le (1-\frac{{T}_{C}}{{T}_{H}})\,\frac{{{\mathscr{T}}}_{R}}{{{\mathscr{T}}}_{R}-{T}_{C}},$$

and is determined again by the apparent temperature of *R* (assumed larger than *T*_*C*_, otherwise the energy extraction is trivial). The above results and comments valid for refrigeration are also applicable in the energy extraction regime. Moreover, *R* is expected to reach a steady state characterised by ${\dot{E}}_{R}=0$ which corresponds to an apparent temperature equal to *ν*_0_[(*ω*_0_ + *ν*_0_)/*T*_*H*_ − *ω*_0_/*T*_*C*_]^−1^ (taking value outside of the interval [*T*_*C*_; *T*_*H*_]). Therefore, one can see that the higher the efficiency, the lower the steady state apparent temperature and the sooner the machine stops extracting energy (and conversely). This effect together with the interplay with bath-induced coherences and correlations in the steady state deserves further investigations.

## Discussion

We explore autonomous machines, a platform naturally adapted to investigate quantum effects in thermodynamic tasks. The existing designs of autonomous thermal machines are broadly extended (from quantum batteries made of single harmonic oscillators to arbitrary system of single energy transition or even discrete spectrum). In order to inspect more deeply perspectives of quantum boosts potentially hidden in previous upper bounds based on the Second Law we establish the actual efficiency of autonomous machines. This enables us to determine the maximal achievable efficiency. Thanks to the apparent temperature introduced in^3^, it takes a simple an physically meaningful expression: the maximal achievable efficiency corresponds to the Carnot bound substituting the usual temperature by the apparent temperature of the quantum battery. Beyond increasing the validity and relevance of the apparent temperature, this happens to be an enlightening tool to understand the role of coherence, many-body correlations, and additional non-thermal characteristics in autonomous machines. Namely, as shown in^3^, coherence and correlations contribute to heat flows together with the populations. Consequently, the usual population balance is modified which results in affecting the apparent temperature. The overall balance on the achievable efficiency is given in (10) and (13), providing conditions for coherence and correlations to be beneficial, but also quantifying the resulting advantages. These results together with the broad validity of our model is expected to be fundamental in the realisation of autonomous thermal machines boosted by quantum and non-thermal effects.

## Methods

### Upper bound from the Second Law

We detail in this paragraph the performances that can be ideally expected from such machines according to the Second Law of Thermodynamics. Under the Markovian approximation and following^59,63,66^ the Second Law applied to the ensemble *SR* interacting with baths *C* and *H* takes the form of Spohn’s inequality^67^,

18 $$\dot{S}({\rho }_{SR})-\frac{{\dot{Q}}_{SR/C}}{{T}_{C}}-\frac{{\dot{Q}}_{SR/H}}{{T}_{H}}\ge 0,$$

where *S*(*ρ*_*SR*_) = −Tr{*ρ*_*SR*_ ln *ρ*_*SR*_} is the von Neumann entropy of the state *ρ*_*SR*_, and *T*_*H*_ and *T*_*C*_ are the temperatures of *H* and *C*, respectively. For *S* in a steady state (as required for a continuous machines^35,47,52,53,66^), we derive from the energy conservation (First Law) and from the Second Law (18) the following upper bound for the efficiency,

19 $$\eta \le \frac{{T}_{C}}{{T}_{H}-{T}_{C}}\,(1+{T}_{H}\frac{\dot{S}({\rho }_{R})}{-\,{\dot{E}}_{R}}),$$

where $${\rho }_{R}\,:\,={{\rm{Tr}}}_{S}{\rho }_{SR}$$.

Interestingly, this expression shows explicitly the decoupling of entropy and energy for non-thermal states. The two usual situations can also be recovered from (19). Namely, when *R* provides energy without any entropy cost, corresponding to $\dot{S}({\rho }_{R})=0$ and usually identified as *work* (Fig. 1, panel a), and when *R* is a thermal bath, where energy and entropy are *bound* by the relation $\dot{S}({\rho }_{R})={\dot{E}}_{R}/{T}_{R}$, which corresponds to the absorption refrigerator (Fig. 1, panel b). In both situations the corresponding Carnot bound is recovered from (19). The present study goes beyond these two usual situations (Fig. 1, panel c) and shows how quantum coherence, many-body correlations, and non-thermal population distribution can be exploited to boost autonomous thermal machines. The upper bound (19) was already derived in^51–53^. However, its attainability is not proven, and as discussed in the main text, there are several reasons to believe that it is not achievable.

### Why dispersive coupling?

We consider the action of the baths *H* and *C* denoted collectively by *B* on the ensemble *SR* and adapt the formalism of Breuer and Petruccione’s book^55^ to derive corrections to the local approach of the master equation of the reduced dynamic of *ρ*_*SR*_^56^. Assuming the bath couplings are weak (*λ*_*C*_ and *λ*_*H*_ much smaller than the inverse of the bath correlation times), we start from the Redfield equation incremented with Markov approximation. This corresponds to Eq. (3.118) in^55^, (setting *ħ* = 1),

20 $$\frac{d}{dt}{\rho }_{SR}^{I}(t)=-\,{\int }_{0}^{\infty }\,ds{{\rm{Tr}}}_{B}[{V}_{SB}^{I}(t),[{V}_{SB}^{I}(t-s),{\rho }_{SR}^{I}(t)\otimes {\rho }_{B}]],$$

where the superscript *I* denotes the interaction picture with respect to *H*_*SR*_ + *H*_*B*_, namely ${\rho }_{SR}^{I}(t)\,:\,={e}^{i{H}_{SR}t}{\rho }_{SR}{e}^{-i{H}_{SR}t}$, ${V}_{SB}^{I}(t)\,:\,={e}^{i({H}_{SR}+{H}_{B})t}{V}_{SB}{e}^{-i({H}_{SR}+{H}_{B})t}$ = ${A}_{S}^{I}(t){\tilde{A}}_{B}(t)$, with ${A}_{S}^{I}(t)\,:\,={e}^{i{H}_{SR}t}{A}_{S}{e}^{-i{H}_{SR}t}$. We introduced the different notation of operators bearing tilde to denote interaction picture *with respect to the free Hamiltonian* of the corresponding subsystems, so that ${\tilde{A}}_{B}(t)\,:\,={e}^{i{H}_{B}t}{A}_{B}{e}^{-i{H}_{B}t}$. In the following we consider that *λ*_*H*_ (*λ*_*C*_) is included in *A*_*H*_ (*A*_*C*_). We define the rate of change of ${\rho }_{SR}^{I}$ only due to the interaction with the bath *j* as

21 $${\dot{\rho }}_{SR{|}_{j}}^{I}(t)\,:\,=-\,{\int }_{0}^{\infty }\,ds{{\rm{Tr}}}_{j}[{V}_{Sj}^{I}(t),[{V}_{Sj}^{I}(t-s),{\rho }_{SR}^{I}(t)\otimes {\rho }_{j}]],$$

where *ρ*_*j*_ denotes the density matrix of the bath *j* and ${V}_{Sj}^{I}(t-s)$ denotes the operator ${V}_{Sj}\,:\,={A}_{S}{A}_{j}$ (with *λ*_*j*_ included within *A*_*j*_) in the interaction picture defined above. One should note that since the baths are assumed to be thermal (and to remain independent), the following equality holds,

22 $${\dot{\rho }}_{SR}^{I}(t)={\dot{\rho }}_{SR{|}_{C}}^{I}(t)+{\dot{\rho }}_{SR{|}_{H}}^{I}(t).$$

We are ultimately interested in the heat flow from the bath *j* = *H*, *C* entering *SR* defined in (1). Injecting the above expression (21) of ${\dot{\rho }}_{SR{|}_{j}}^{I}$ in (1) one obtains,

23 $${\dot{Q}}_{SR/j}\,:\,=-\,{\int }_{0}^{\infty }\,ds{\langle {\tilde{A}}_{j}(s){A}_{j}\rangle }_{{\rho }_{j}}{{\rm{Tr}}}_{SR}{\rho }_{SR}^{I}(t)[{H}_{SR},{A}_{S}^{I}(t)]{A}_{S}^{I}(t-s)+c.\,c.$$

In the reminder of the Supplementary Material (as well as in the main text), we use the notation ${\langle {\mathscr{O}}\rangle }_{\rho }$ to denote the expectation value of the operator ${\mathscr{O}}$ in the state *ρ*. Interestingly, the commutator appearing in the above expression can be calculated as follow,

24 $$\begin{array}{rcl}[{H}_{SR},{A}_{S}^{I}(t)] & = & {e}^{i{H}_{SR}t}[{H}_{SR},{A}_{S}]{e}^{-i{H}_{SR}t}\\  & = & {e}^{i{H}_{SR}t}([{H}_{S},{A}_{S}]+g{A}_{R}[{N}_{S},{A}_{S}]){e}^{-i{H}_{SR}t}.\end{array}$$

Then, it appears that if *N*_*S*_ (the observable realising the coupling between *S* and *R*) commutes with *A*_*S*_ (the observable involved in the coupling between *S* and the baths), the heat flows between *SR* and the baths do not involve actively *R*. In other words one would have ${\dot{Q}}_{SR/j}={\dot{Q}}_{S/j}$, meaning that the energy contained in *R* and the correlations *V*_*SR*_ is constant, which does not seem very promising for a thermal machine powered by *R*. More generally, if [*N*_*S*_, *A*_*S*_] = $\kappa {\mathbb{I}}$, where ${\mathbb{I}}$ stands for the identity and *κ* is a complex number, the heat flow becomes

25 $${\dot{Q}}_{SR/j}={\dot{Q}}_{S/j}-g\kappa \,{\int }_{0}^{\infty }\,ds{\langle {\tilde{A}}_{j}(s){A}_{j}\rangle }_{{\rho }_{j}}{{\rm{Tr}}}_{SR}{\rho }_{SR}^{I}(t){A}_{R}^{I}(t){A}_{S}^{I}(t-s)+c.\,c.$$

In the second term of the right-hand side, contributions of first and second order in *g* are rapidly oscillating (at combinations of frequencies *ω*_0_ and *ν*) so that they cancel out on average (secular approximation). Consequently, we obtain ${\dot{Q}}_{SR/j}={\dot{Q}}_{S/j}+{\mathscr{O}}({g}^{3}/|\nu {|}^{3})$, which again is problematic for a thermal machine. For each kind of system *S* (in particular for *S* a two-level system or a harmonic oscillator), there are several possible choices for *N*_*S*_ to avoid $[{N}_{S},{A}_{S}]=\kappa {\mathbb{I}}$. However, the only choice that avoid the problem simultaneously for both harmonic oscillators and two-level systems (and therefore for any other systems) is *N*_*S*_ = *αH*_*S*_ where *α* is a real number. For this reason we choose such coupling (dispersive coupling) between *S* and *R*.

### Expression of the bath dissipative operators

The derivation of the reduced dynamics of *SR* (following^55^) requires the decomposition of ${A}_{S}^{I}(t)$ in the form ${A}_{S}^{I}(t)={\sum }_{{\rm{\Omega }}}\,{e}^{-i{\rm{\Omega }}t}{\mathscr{A}}({\rm{\Omega }})$ where the operators ${\mathscr{A}}({\rm{\Omega }})$ are eigenoperators of *H*_*SR*_ (also called ladder operators) verifying the relation $[{H}_{SR},{\mathscr{A}}({\rm{\Omega }})]=-\,{\rm{\Omega }}{\mathscr{A}}({\rm{\Omega }})$, and the sum runs over an ensemble of frequencies Ω generated by combinations of the transition frequencies *ω* and *ν* of *S* and *R*, respectively. The difficulty here comes from the interaction term *V*_*SR*_ which turns the Hamiltonian *H*_*SR*_ non-diagonalisable. As a consequence such eigenoperator decomposition is only approximately accessible through an expansion in term of the coupling strength *g* between *S* and *R*. We detail in the following the main steps leading to such decomposition. We start with

26 $$\begin{array}{rcl}{A}_{S}^{I}(t) & = & {e}^{i{H}_{SR}t}{A}_{S}{e}^{-i{H}_{SR}t}\\  & = & \exp \,\{ig\bar{{\mathscr{T}}}\,{\int }_{0}^{t}\,du{N}_{S}{\tilde{A}}_{R}(u)\}{e}^{i{H}_{S}t}{A}_{S}{e}^{-i{H}_{S}t}\,\exp \,\{\,-ig{\mathscr{T}}\,{\int }_{0}^{t}\,du{N}_{S}{\tilde{A}}_{R}(u)\},\end{array}$$

where ${\mathscr{T}}$ and $\bar{{\mathscr{T}}}$ denote the time ordering and anti-chronological ordering operators respectively (and ${\tilde{A}}_{R}(u)\,:\,={e}^{i{H}_{R}u}{A}_{R}{e}^{-i{H}_{R}u}$). For the system *X* = *S*, *R*, we decompose *A*_*X*_ in a sum of eigenoperators *A*_*X*_(*ν*)^55^ as introduced after (2), so that ${\tilde{A}}_{X}(u)={\sum }_{\nu \in { {\mathcal E} }_{X}}\,{A}_{X}(\nu ){e}^{-i\nu u}$, the sum running over the authorised transition frequencies of *X*, denoted by the ensemble ${ {\mathcal E} }_{X}$. The eigenoperators verify [*H*_*X*_, *A*_*X*_(*ν*)] = −*νA*_*X*_(*ν*) and ${A}_{R}(\,-\,\nu )={A}_{R}^{\dagger }(\nu )$ (Hermicity of *A*_*R*_) which implies that if *ν* belongs to ${ {\mathcal E} }_{R}$ then, −*ν* too.

To obtain the final expression of the heat flow (2) we restrict *S* to be a two-level system or a harmonic oscillator but for now we continue with the general case as simplify the notations. Then, assuming $g/|\nu |\ll 1$ for all $\nu \in { {\mathcal E} }_{R}$ and retaining only contributions up to second order in *g*/|*ν*|, ${A}_{S}^{I}(t)$ can be rewritten as

27 $$\begin{array}{rcl}{A}_{S}^{I}(t) & = & \sum _{\omega \in { {\mathcal E} }_{S}}\,{e}^{-i\omega t}\,\exp \,\{ig\bar{{\mathscr{T}}}\,{\int }_{0}^{t}\,du{N}_{S}{\tilde{A}}_{R}(u)\}\\  &  & \times \,{A}_{S}(\omega )\,\exp \,\{\,-ig{\mathscr{T}}\,{\int }_{0}^{t}\,du{N}_{S}{\tilde{A}}_{R}(u)\}\\  & = & \sum _{\omega \in { {\mathcal E} }_{S}}\,{e}^{-i\omega t}{A}_{S}(\omega )\,\{1+g\alpha \omega \,\sum _{\nu \in { {\mathcal E} }_{R}}\,{A}_{R}(\nu )\tfrac{{e}^{-i\nu t}-1}{\nu }\\  &  & -\,{g}^{2}\,\sum _{{\nu }_{1}\ne -{\nu }_{2}\in {E}_{R}}\,[{\alpha }^{2}{\omega }^{2}{A}_{R}({\nu }_{2}){A}_{R}({\nu }_{1})\\  &  & -\,\alpha \omega {N}_{S}[{A}_{R}({\nu }_{2}),{A}_{R}({\nu }_{1})]]\,[\tfrac{{e}^{-i{\nu }_{1}t}}{{\nu }_{1}{\nu }_{2}}-\tfrac{{e}^{-i({\nu }_{1}+{\nu }_{2})t}}{{\nu }_{2}({\nu }_{1}+{\nu }_{2})}-\tfrac{1}{{\nu }_{1}({\nu }_{1}+{\nu }_{2})}]\\  &  & -\,{g}^{2}\,\sum _{\nu \in { {\mathcal E} }_{R}}\,[{\alpha }^{2}{\omega }^{2}{A}_{R}^{\dagger }(\nu ){A}_{R}(\nu )\\  &  & -\,\alpha \omega {N}_{S}[{A}_{R}^{\dagger }(\nu ),{A}_{R}(\nu )]]\,[\tfrac{1-i\nu t-{e}^{-i\nu t}}{{\nu }^{2}}]+{\mathscr{O}}({g}^{3}/|\nu {|}^{3})\}.\end{array}$$

The operator *N*_*S*_ being proportional to *H*_*S*_, the commutator of *N*_*S*_ with *A*_*S*_(*ω*) is [*N*_*S*_, *A*_*S*_(*ω*)] = −*αωA*_*S*_(*ω*). Rearranging (27) we finally have a form resembling the eigenoperator decomposition we are looking for,

28 $${A}_{S}^{I}(t)=\sum _{\omega \in { {\mathcal E} }_{S}}\,[{\mathscr{A}}(\omega )+{\mathscr{C}}(\omega )t]\,{e}^{-i\omega t}+\sum _{\omega \in { {\mathcal E} }_{S},\nu \in { {\mathcal E} }_{R}}\,{\mathscr{A}}(\omega +\nu ){e}^{-i(\omega +\nu )t}+{\mathscr{O}}(\frac{{g}^{2}}{{\nu }^{2}}),$$

where for $$\omega \in { {\mathcal E} }_{S}$$,

29 $$\begin{array}{rcl}{\mathscr{A}}(\omega ) & = & {A}_{S}(\omega )[1-\sum _{\nu \in { {\mathcal E} }_{R}}\,\frac{g\alpha \omega }{\nu }{A}_{R}(\nu )+\,{g}^{2}\,\sum _{{\nu }_{1}\ne -{\nu }_{2}\in { {\mathcal E} }_{R}}\,\frac{1}{{\nu }_{1}({\nu }_{1}+{\nu }_{2})}\\  &  & \times \,[{\alpha }^{2}{\omega }^{2}{A}_{R}({\nu }_{2}){A}_{R}({\nu }_{1})-\,\alpha \omega {N}_{S}[{A}_{R}({\nu }_{2}),{A}_{R}({\nu }_{1})]]\\  &  & -\,{g}^{2}\,\sum _{\nu \in { {\mathcal E} }_{R}}\,\frac{{\alpha }^{2}{\omega }^{2}}{{\nu }^{2}}{A}_{R}^{\dagger }(\nu ){A}_{R}(\nu )+{\mathscr{O}}(\frac{{g}^{3}}{|\nu {|}^{3}})],\end{array}$$

30 $${\mathscr{C}}(\omega )=i{g}^{2}{\alpha }^{2}[{H}_{S}^{2},{A}_{S}(\omega )]\,\sum _{\nu \in { {\mathcal E} }_{R}}\,\frac{1}{\nu }[{A}_{R}^{\dagger }(\nu ),{A}_{R}(\nu )],$$

and for $\omega \in { {\mathcal E} }_{S}$ and $\nu \in { {\mathcal E} }_{R}$,

31 $${\mathscr{A}}(\omega +\nu )=\frac{g\alpha \omega }{\nu }{A}_{S}(\omega ){A}_{R}(\nu )+{\mathscr{O}}(\frac{{g}^{2}}{{\nu }^{2}}).$$

One can verify the following identities, ${\mathscr{A}}(\,-\,\omega )={{\mathscr{A}}}^{\dagger }(\omega )$, ${\mathscr{C}}(\,-\,\omega )={{\mathscr{C}}}^{\dagger }(\omega )$, and ${\mathscr{A}}(\,-\,\omega -\nu )={{\mathscr{A}}}^{\dagger }(\omega +\nu )$. Terms of second order in *g*/|*ν*| have already been dropped in Eqs (28) and (31) since they generate in the master equation only terms of order 3 and 4. The injection of the decomposition (28) into Eq. (20) brings the following master equation which takes into account the *SR* coupling up to second order processes,

32 $$\begin{array}{ccc}{\dot{\rho }}_{SR}^{I} & = & \sum _{\omega \in {{\mathscr{E}}}_{S}}\,{\rm{\Gamma }}(\omega )[{\mathscr{A}}(\omega ){\rho }_{SR}^{I}{{\mathscr{A}}}^{\dagger }(\omega )-\,{{\mathscr{A}}}^{\dagger }(\omega ){\mathscr{A}}(\omega ){\rho }_{SR}^{I}]+{\rm{h}}.\,{\rm{c}}.\\  &  & +\,\sum _{\omega \in {{\mathscr{E}}}_{S},\nu \in {{\mathscr{E}}}_{R}}\,{\rm{\Gamma }}(\omega +\nu )\,[{\mathscr{A}}(\omega +\nu ){\rho }_{SR}^{I}{{\mathscr{A}}}^{\dagger }(\omega +\nu )\\  &  & -\,{{\mathscr{A}}}^{\dagger }(\omega +\nu )\,{\mathscr{A}}(\omega +\nu ){\rho }_{SR}^{I}]+{\rm{h}}.\,{\rm{c}}.\\  &  & +\,{{\rm{\Lambda }}}_{t}({\rho }_{SR}^{I}),\end{array}$$

where the secular approximation^55^ was performed for the terms involving operators ${{\mathscr{A}}}_{S}$, leading to the first two lines of (32). However, for terms involving ${\mathscr{C}}(\omega )t$ the secular approximation is not valid due to the time dependence. The resulting map is denoted by Λ_*t*_ and is given by

33 $$\begin{array}{rcl}{{\rm{\Lambda }}}_{t}({\rho }_{SR}^{I}) & = & -i\,\sum _{\omega \in { {\mathcal E} }_{S}}\,{\partial }_{\omega }{\rm{\Gamma }}(\omega )[{A}_{S}^{\dagger }(\omega ){\mathscr{C}}(\omega ){\rho }_{SR}^{I}-{\mathscr{C}}(\omega ){\rho }_{SR}^{I}{A}_{S}^{\dagger }(\omega )]+{\rm{h}}.\,{\rm{c}}.\\  &  & +\,\sum _{\omega ,\omega ^{\prime} \in { {\mathcal E} }_{S}}\,{\rm{\Gamma }}(\omega ){e}^{i(\omega ^{\prime} -\omega )t}t[{\mathscr{C}}(\omega ){\rho }_{SR}^{I}{A}_{S}^{\dagger }(\omega ^{\prime} )\\  &  & -\,{A}_{S}^{\dagger }(\omega ^{\prime} ){\mathscr{C}}(\omega ){\rho }_{SR}^{I}+{A}_{S}(\omega ){\rho }_{SR}^{I}{{\mathscr{C}}}^{\dagger }(\omega ^{\prime} )\\  &  & -\,{{\mathscr{C}}}^{\dagger }(\omega ^{\prime} ){A}_{S}(\omega ){\rho }_{SR}^{I}]+{\rm{h}}.\,{\rm{c}}.\\  &  & +\,{\mathscr{O}}(\frac{{g}^{3}}{|\nu {|}^{3}}),\end{array}$$

where the complex function Γ(*ω*) is defined by ${\rm{\Gamma }}(\omega )\,:\,={{\rm{\Gamma }}}_{C}(\omega )+{{\rm{\Gamma }}}_{H}(\omega )$ and

34 $${{\rm{\Gamma }}}_{j}(\omega )\,:\,={\int }_{0}^{\infty }\,ds{e}^{i\omega s}{{\rm{Tr}}}_{j}{\rho }_{j}{A}_{j}^{I}(s){A}_{j}^{I}(0),$$

is of second order in the coupling coefficient *λ*_*j*_, for *j* = *C*, *H*. We also define the spectral density of the bath *j*,

35 $${G}_{j}(\omega )\,:\,={{\rm{\Gamma }}}_{j}(\omega )+{{\rm{\Gamma }}}_{j}^{\ast }(\omega ),$$

and, $G(\omega )\,:\,={G}_{H}(\omega )+{G}_{C}(\omega )$. We mention the following useful identity valid for thermal baths (*k*_*B*_ = 1),

36 $${G}_{j}(\,-\,\omega )={e}^{-\omega /{T}_{j}}{G}_{j}(\omega ).$$

One should note the presence of the factor *t* in the second line of (33). Its linear growth in time can end up dominating the low-order terms. We show in the following that its contribution to the energy flow cancels out. However, one could wonder what happen if similar factors appear in higher order terms. Firstly, similar cancellation may happen (however showing it systematically is a challenging task). Secondly, one can easily show that terms of third order grow at most as $\frac{{g}^{3}}{|\nu {|}^{3}}|\nu |t$. Assuming the coupling between *S* and *R* smaller than the coupling with the baths, $g\ll {G}_{C}({\omega }_{0})$, such third order terms might become significant only for times much larger than ${\tau }_{R}\,:\,={({G}_{C}({\omega }_{0})\frac{{g}^{2}}{|\nu {|}^{2}})}^{-1}$ which is the evolution timescale of *R* and of the energy flows. Similar reasoning can be repeated for higher orders. As a consequence, the contribution from higher orders might become relevant only after *R* delivered (or stored) a significant amount of energy. Finally, on a more physical ground, one can observe that higher order terms in (27) should be relevant if *S* and *R* were interacting in a close dynamics, without the presence of the baths. However, the decohering action of the baths limits the coherence of *S* and therefore limits also the correlation time between *S* and *R*. For weak coupling $g\ll {\lambda }_{C},\,{\lambda }_{H}$, this dissipates high order processes between *S* and *R*. Under these conditions, we limit ourselves to expansions of second order in *g*/*ν*_0_. Considering that all transition energies *ν* of *R* are of same order of magnitude, we write ${\mathscr{O}}(\frac{{g}^{3}}{{\nu }_{0}^{3}})$ instead of ${\mathscr{O}}(\frac{{g}^{3}}{|\nu {|}^{3}})$ (valid also for Section “Results”).

From (32) and the decomposition of Γ(*ω*) in contributions from each bath one can rewrite the above master equation as

37 $${\dot{\rho }}_{SR}^{I}=({ {\mathcal L} }_{C}+{ {\mathcal L} }_{H}){\rho }_{SR}^{I},$$

where ${ {\mathcal L} }_{j}$ is the dissipative operator of the bath *j*, obtained from (32) and (33) by substituting Γ(*ω*) by Γ_*j*_(*ω*).

### Heat flows

We define the heat flow from the bath *j* = *C*, *H* to the system *S*, *R*, or *SR*, denoted by *X* in the following, as

38 $${\dot{Q}}_{X/j}\,:\,={{\rm{Tr}}}_{SR}{ {\mathcal L} }_{j}{\rho }_{SR}^{I}{H}_{X}.$$

When *X* = *SR* we recover the definition (1). The heat flows are computed by injecting the expression of the bath dissipative operators in the above heat flow definition (38). The calculation leading to (2) is not complex but very cumbersome. The full detail can be found in the Supplementary Information. The main idea guiding the derivation is that *S* is brought in a time interval of the order ${\tau }_{es}\,:\,={[{G}_{C}({\omega }_{0})]}^{-1}$ to a thermal state at temperature *T*_*C*_ (up to corrections of order *g*/*ν*_0_) due to its resonant and direct interaction with *C*. This timescale *τ*_*es*_ is much smaller than ${\tau }_{R}={[{G}_{C}({\omega }_{0}){g}^{2}/{\nu }_{0}^{2}]}^{-1}$ the evolution timescale of *R* and of the energy flows. Then, it is legitimate to approximate ${\rho }_{S}^{I}(t)$ by a thermal state at temperature *T*_*C*_ in terms of second order in *g*/*ν*_0_. However, the expectation value ${\langle {A}_{S}({\omega }_{0}){A}_{S}^{\dagger }({\omega }_{0})\rangle }_{{\rho }_{S}^{I}(t)}$ appears in the expansion of the expression of the heat flow (38) and is not of second order in *g*/*ν*_0_. Substituting ${\rho }_{S}^{I}(t)$ by the thermal state at the temperature *T*_*C*_ is therefore not legitimate. We determine the expectation value ${\langle {A}_{S}({\omega }_{0}){A}_{S}^{\dagger }({\omega }_{0})\rangle }_{{\rho }_{S}^{I}(t)}$ up to second order in *g*/*ν*_0_ when *S* is a harmonic oscillator or a two-level system (see Supplementary Information). When *S* is a harmonic oscillator, the resulting expression for the heat flow from the cold bath *C* is (for $t\gg {\tau }_{es}$)

39 $$\begin{array}{rcl}{\dot{Q}}_{SR/C} & = & {\omega }_{0}\tfrac{{g}^{2}{\alpha }^{2}{\omega }_{0}^{2}}{{\nu }_{0}^{2}}\tfrac{{G}_{C}({\omega }_{0}){G}_{H}({\omega }_{0}+{\nu }_{0})}{{G}_{C}({\omega }_{0})-{G}_{C}(-{\omega }_{0})}({e}^{-\tfrac{{\omega }_{0}}{{T}_{C}}}{\langle {A}_{R}^{\dagger }({\nu }_{0}){A}_{R}({\nu }_{0})\rangle }_{{\rho }_{R}^{I}}\\  &  & -\,{e}^{-\tfrac{{\omega }_{0}+{\nu }_{0}}{{T}_{H}}}{\langle {A}_{R}({\nu }_{0}){A}_{R}^{\dagger }({\nu }_{0})\rangle }_{{\rho }_{R}^{I}})+{\mathscr{O}}({g}^{3}/{\nu }_{0}^{3}).\end{array}$$

For a two-level system the expression is the same expect for the denominator of the pre-factor which is change to *G*_*C*_(*ω*_0_) + *G*_*C*_(−*ω*_0_) instead of *G*_*C*_(*ω*_0_) − *G*_*C*_(−*ω*_0_). For both systems the following identity holds,

40 $${\dot{E}}_{R}=\frac{{\nu }_{0}}{{\omega }_{0}+{\nu }_{0}}{\dot{Q}}_{SR/H}+{\mathscr{O}}(\frac{{g}^{3}}{{\nu }_{0}^{3}})=-\,\frac{{\nu }_{0}}{{\omega }_{0}}{\dot{Q}}_{SR/C}+{\mathscr{O}}(\frac{{g}^{3}}{{\nu }_{0}^{3}}).$$

Furthermore, the rate of variation of *E*_*S*_ is ${\dot{E}}_{S}={\mathscr{O}}({g}^{3}/{\nu }_{0}^{3})$ for $t\gg {\tau }_{es}$ for both systems.

## Supplementary information

Supplemental Material

## References

[1] Mohammady MH (2018). Low-control and robust quantum refrigerator and applications with electronic spins in diamond. Phys. Rev. A.

[2] Silveri M, Grabert H, Masuda S, Tan KY, Mottonen M (2017). Theory of quantum-circuit refrigeration by photon-assisted electron tunneling. Phys. Rev. B.

[3] Latune, C. L., Sinayskiy, I. & Petruccione, F. Apparent temperature: demystifying the relation between quantum coherence, correlations, and heat flows. *Quantum Sci*. *Technol* (2018).

[4] Scully MO, Zubairy MS, Agarwal GS, Walther H (2003). Extracting Work from a Single Heat Bath via Vanishing Quantum Coherence. Science.

[5] Scully MO (2002). Extracting Work from a Single Heat Bath via Vanishing Quantum Coherence II: Microscopic Model. AIP Conference Proceedings.

[6] Brandner K, Bauer M, Seifert U (2017). Universal Coherence-Induced Power Losses of Quantum Heat Engines in Linear Response. Phys. Rev. Lett..

[7] Mehta V, Johal RS (2017). Quantum Otto engine with exchange coupling in the presence of level degeneracy. Phys. Rev. E.

[8] Türkpençe D, Müstecaplıoǧlu ÖE (2016). Quantum fuel with multilevel atomic coherence for ultrahigh specific work in a photonic Carnot engine. Phys. Rev. E.

[9] Türkpençe D, Altintas F, Paternostro M, Müstecaplıoǧlu ÖE (2017). A photonic Carnot engine powered by a spin-star network. EPL.

[10] Zhang T, Liu W-T, Chen P-X, Li C-Z (2007). Four-level entangled quantum heat engines. Phys. Rev. A.

[11] Wang H, Liu S, He J (2009). Thermal entanglement in two-atom cavity QED and the entangled quantum Otto engine. Phys. Rev. E.

[12] Dillenschneider R, Lutz E (2009). Energetics of quantum correlations. EPL.

[13] Hardal AÜC, Müstecaplıoǧlu ÖE (2015). Superradiant Quantum Heat Engine. Scientific Reports.

[14] Altintas F, Hardal AÜC, Müstecaplıoǧlu ÖE (2014). Quantum correlated heat engine with spin squeezing. Phys. Rev. E.

[15] Altintas F, Hardal AÜC, Müstecaplıoǧlu ÖE (2015). Rabi model as a quantum coherent heat engine: From quantum biology to superconducting circuits. Phys. Rev. A.

[16] Hardal AÜC, Paternostro M, Müstecaplıoǧlu ÖE (2018). Phase-space interference in extensive and nonextensive quantum heat engines. Phys. Rev. E.

[17] Doyeux P, Leggio B, Messina R, Antezza M (2016). Quantum thermal machine acting on a many-body quantum system: Role of correlations in thermodynamic tasks. Phys. Rev. E.

[18] Li H (2014). Quantum coherence rather than quantum correlations reflect the effects of a reservoir on a system’s work capability. Phys. Rev. E.

[19] Jaramillo J, Beau M, Campo AD (2016). Quantum supremacy of many-particle thermal machines. New J. Phys..

[20] Mueller, M. P. Correlating thermal machines and the second law at the nanoscale. *arXiv*: *1707*.*03451* [*cond*-*mat*, *physics*:*quant*-*ph*] (2017).

[21] Gardas B, Deffner S (2015). Thermodynamic universality of quantum Carnot engines. Phys. Rev. E.

[22] Leggio B, Antezza M (2016). Otto engine beyond its standard quantum limit. Phys. Rev. E.

[23] Abah O, Lutz E (2014). Efficiency of heat engines coupled to nonequilibrium reservoirs. EPL.

[24] Roßnagel J, Abah O, Schmidt-Kaler F, Singer K, Lutz E (2014). Nanoscale Heat Engine Beyond the Carnot Limit. Phys. Rev. Lett..

[25] Manzano G, Galve F, Zambrini R, Parrondo JMR (2016). Entropy production and thermodynamic power of the squeezed thermal reservoir. Phys. Rev. E.

[26] Huang XL, Wang T, Yi XX (2012). Effects of reservoir squeezing on quantum systems and work extraction. Phys. Rev. E.

[27] Niedenzu W, Gelbwaser-Klimovsky D, Kurizki G (2015). Performance limits of multilevel and multipartite quantum heat machines. Phys. Rev. E.

[28] Gelbwaser-Klimovsky D, Niedenzu W, Brumer P, Kurizki G (2015). Power enhancement of heat engines via correlated thermalization in a three-level “working fluid”. Scientific Reports.

[29] Clivaz, F. *et al*. Unifying paradigms of quantum refrigeration: how resource-control determines fundamental limits. *arXiv*: *1710*.*11624* [*cond*-*mat*, *physics*:*quant*-*ph*] (2017).

[30] Uzdin R, Levy A, Kosloff R (2015). Equivalence of Quantum Heat Machines, and Quantum-Thermodynamic Signatures. Phys. Rev. X.

[31] Brunner N (2014). Entanglement enhances cooling in microscopic quantum refrigerators. Phys. Rev. E.

[32] Brask JB, Brunner N (2015). Small quantum absorption refrigerator in the transient regime: Time scales, enhanced cooling, and entanglement. Phys. Rev. E.

[33] Mitchison MT, Woods MP, Prior J, Huber M (2015). Coherence-assisted single-shot cooling by quantum absorption refrigerators. New J. Phys..

[34] Palao JP, Kosloff R, Gordon JM (2001). Quantum thermodynamic cooling cycle. Phys. Rev. E.

[35] Levy A, Kosloff R (2012). Quantum Absorption Refrigerator. Phys. Rev. Lett..

[36] Venturelli D, Fazio R, Giovannetti V (2013). Minimal Self-Contained Quantum Refrigeration Machine Based on Four Quantum Dots. Phys. Rev. Lett..

[37] Correa LA, Palao JP, Adesso G, Alonso D (2013). Performance bound for quantum absorption refrigerators. Phys. Rev. E.

[38] Correa LA, Palao JP, Alonso D, Adesso G (2014). Quantum-enhanced absorption refrigerators. Scientific Reports.

[39] Correa LA, Palao JP, Adesso G, Alonso D (2014). Optimal performance of endoreversible quantum refrigerators. Phys. Rev. E.

[40] Correa LA (2014). Multistage quantum absorption heat pumps. Phys. Rev. E.

[41] Hofer PP (2016). Autonomous quantum refrigerator in a circuit QED architecture based on a Josephson junction. Phys. Rev. B.

[42] Mitchison MT, Huber M, Prior J, Woods MP, Plenio MB (2016). Realising a quantum absorption refrigerator with an atom-cavity system. Quantum Sci. Technol..

[43] He Z-C, Huang X-Y, Yu C-S (2017). Enabling the self-contained refrigerator to work beyond its limits by filtering the reservoirs. Phys. Rev. E.

[44] Mari A, Eisert J (2012). Cooling by Heating: Very Hot Thermal Light Can Significantly Cool Quantum Systems. Phys. Rev. Lett..

[45] Linden N, Popescu S, Skrzypczyk P (2010). How Small Can Thermal Machines Be? The Smallest Possible Refrigerator. Phys. Rev. Lett..

[46] Skrzypczyk P, Brunner N, Linden N, Popescu S (2011). The smallest refrigerators can reach maximal efficiency. J. Phys. A: Math. Theor..

[47] Kosloff R, Levy A (2014). Quantum Heat Engines and Refrigerators: Continuous Devices. Annu. Rev. Phys. Chem..

[48] Maslennikov, G. *et al*. Quantum Absorption Refrigerator with Trapped Ions. In *2017 European Conference on Lasers and Electro*-*Optics and European Quantum Electronics Conference* (*2017*) (Optical Society of America, 2017).

[49] Du J-Y, Zhang F-L (2018). Nonequilibrium quantum absorption refrigerator. New J. Phys..

[50] Tonner F, Mahler G (2005). Autonomous quantum thermodynamic machines. Phys. Rev. E.

[51] Boukobza E, Ritsch H (2013). Breaking the Carnot limit without violating the second law: A thermodynamic analysis of off-resonant quantum light generation. Phys. Rev. A.

[52] Gelbwaser-Klimovsky D, Kurizki G (2014). Heat-machine control by quantum-state preparation: From quantum engines to refrigerators. Phys. Rev. E.

[53] Gelbwaser-Klimovsky D, Kurizki G (2015). Work extraction from heat-powered quantized optomechanical setups. Scientific Reports.

[54] Geusic JE, Schulz-DuBios EO, Scovil HED (1967). Quantum Equivalent of the Carnot Cycle. Phys. Rev..

[55] Breuer, H.-P. & Petruccione, F. *The Theory of Open Quantum Systems* (Oxford University Press, 2007).

[56] Trushechkin AS, Volovich IV (2016). Perturbative treatment of inter-site couplings in the local description of open quantum networks. EPL.

[57] Levy A, Kosloff R (2014). The local approach to quantum transport may violate the second law of thermodynamics. EPL.

[58] González JO (2017). Testing the Validity of the ‘Local’ and ‘Global’ GKLS Master Equations on an Exactly Solvable Model. Open Syst. Inf. Dyn..

[59] Alicki R (1979). The quantum open system as a model of the heat engine. J. Phys. A: Math. Gen..

[60] Ghosh A, Latune CL, Davidovich L, Kurizki G (2017). Catalysis of heat-to-work conversion in quantum machines. PNAS.

[61] Niedenzu W, Mukherjee V, Ghosh A, Kofman AG, Kurizki G (2018). Quantum engine efficiency bound beyond the second law of thermodynamics. Nature Communications.

[62] Santos, J. P., Céleri, L. C., Landi, G. T. & Paternostro, M. The role of quantum coherence in non-equilibrium entropy production. *arXiv*:*1707*.*08946* [*quant*-*ph*] (2017).

[63] Strasberg P, Schaller G, Brandes T, Esposito M (2017). Quantum and Information Thermodynamics: A Unifying Framework Based on Repeated Interactions. Phys. Rev. X.

[64] Dicke RH (1954). Coherence in Spontaneous Radiation Processes. Phys. Rev..

[65] Gross M, Haroche S (1982). Superradiance: An essay on the theory of collective spontaneous emission. Physics Reports.

[66] Kosloff R (2013). Quantum Thermodynamics: A Dynamical Viewpoint. Entropy.

[67] Spohn H (1978). Entropy production for quantum dynamical semigroups. Journal of Mathematical Physics.

