# Supplementary material for: Quantum coherence, many-body correlations, and non-thermal effects for autonomous thermal machines
Source: Sci Rep. 2019 Feb 28;9:3191. doi: 10.1038/s41598-019-39300-4 (PMC6395647; doi:10.1038/s41598-019-39300-4)
Supplement: Supplementary file 1 — Supplemental Material [file 41598_2019_39300_MOESM1_ESM.pdf]

Quantum coherence, many-body correlations, and non-thermal effects for autonomous thermal machines
Supplemental Material

C.L. Latune[1], I. Sinayskiy[1,2], F. Petruccione[1,21]

[1]*Quantum Research Group, School of Chemistry and Physics, University of KwaZulu-Natal, Durban, KwaZulu-Natal, 4001, South Africa*

[2]*National Institute for Theoretical Physics (NITheP), KwaZulu-Natal, 4001, South Africa*

(Dated: December 5, 2018)

I. GENERAL EXPRESSION OF THE HEAT FLOWS

A. Expression of $\dot{Q}_{SR/j}$

The heat flow from the bath $j = C, H$ is defined in the main text as $\dot{Q}_{SR/j} := \mathrm{Tr}_{SR}\mathcal{L}_j\rho_{SR}^I H_{SR}$. The expression announced in the main text is obtained by inserting the expression of the dissipative operator $\mathcal{L}_j$ in the above definition. One recurrent approximation (sometimes called the adiabatic approximation), is to consider that S is in a thermal state at temperature T_C denoted by ρ_S^{eq} due to the resonant and continuous contact with the cold bath C. Corrective terms to this approximation are of order $g/|\nu|$ and are time-dependent. However, assuming that $g \ll \lambda_C$, S remains approximately in a thermal state at all times, ensuring therefore that these corrective terms remains very small. As a consequence, when dealing with a term of second order in $g/|\nu|$ we approximate ρ_S^I by ρ_S^{eq}. By contrast, when dealing with terms of lower order, such approximation is not valid since we want a final result taking into account up to second order in g/ν. We mention the following identity used throughout this Section,

$$A_S(\omega)\rho_S^{eq} = e^{-\omega/T_C}\rho_S^{eq}A_S(\omega), \tag{1}$$

derived from

$$\begin{aligned} e^{H_S/T_C}A_S(\omega)e^{-H_S/T_C} &= \sum_{n=0}^{\infty}\frac{1}{n!}\frac{1}{T_C^n}Ad_{H_S}^n A_S(\omega) \\ &= \sum_{n=0}^{\infty}\frac{1}{n!}\frac{1}{T_C^n}(-\omega)^n A_S(\omega) \\ &= e^{-\omega/T_C}A_S(\omega), \end{aligned} \tag{2}$$

where $Ad_M^n N := [M, Ad_M^{n-1}N]$ and $Ad_M^0 N = N$. We recall that the ladder operators are such that $[H_S, A_S(\omega)] = -\omega A_S(\omega)$ so that $Ad_{H_S}^n A_S(\omega) = (-\omega)^n A_S(\omega)$.

Starting with

$$\begin{aligned} \dot{Q}_{SR/j} &= \mathrm{Tr}\{\mathcal{L}_j\rho_{SR}^I H_{SR}\} \\ &= \sum_{\omega\in\mathcal{E}_S}\Gamma_j(\omega)\mathrm{Tr}\{\rho_{SR}^I[\mathcal{A}^\dagger(\omega), H_{SR}]\mathcal{A}(\omega)\} + c.c. && (3) \\ &\quad + \sum_{\omega\in\mathcal{E}_S,\nu\in\mathcal{E}_R}\Gamma_j(\omega+\nu)\mathrm{Tr}\{\rho_{SR}^I[\mathcal{A}^\dagger(\omega+\nu), H_{SR}]\mathcal{A}(\omega+\nu)\} + c.c && (4) \\ &\quad + \mathrm{Tr}\{\Lambda_{j,t}(\rho_{SR}^I)H_{SR}\}, && (5) \end{aligned}$$

we provide in the following the contribution from each of the three terms present in the above expression of the heat flow.

1. Contribution from the term (3)

The commutator $[\mathcal{A}^\dagger(\omega), H_{SR}]$ gives

$$[\mathcal{A}^\dagger(\omega), H_{SR}] = [\mathcal{A}^\dagger(\omega), H_S] + [\mathcal{A}^\dagger(\omega), H_R] + [\mathcal{A}^\dagger(\omega), V_{SR}] \tag{6}$$

Using the expression of $\mathcal{A}(\omega)$ given in Method we find

$$[\mathcal{A}^\dagger(\omega), H_S] = -\alpha\omega\mathcal{A}^\dagger, \tag{7}$$

$$[\mathcal{A}^\dagger(\omega), H_R] = -g\alpha\omega A_S^\dagger(\omega)A_R\left(1+\sum_{\nu\in\mathcal{E}_R}\frac{g\alpha\omega}{\nu}A_R(\nu)\right) + gA_S^\dagger(\omega)N_S\sum_{\nu\in\mathcal{E}_R}\frac{g\alpha\omega}{\nu}[A_R(\nu), A_R] + \mathcal{O}\left(\frac{g^3}{\nu^3}\right), \tag{8}$$

and,

$$\begin{aligned}[\mathcal{A}^\dagger(\omega), V_{SR}] = g\alpha\omega A_S^\dagger(\omega)A_R + g^2A_S^\dagger(\omega)\sum_{\nu\in\mathcal{E}_R}\frac{1}{\nu}\left(\alpha^2\omega^2A_RA_R(\nu) + \alpha\omega N_S[A_R, A_R(\nu)]\right)\\ -g^2A_S^\dagger(\omega)\sum_{\nu\in\mathcal{E}_R}\frac{1}{\nu}\left(\alpha^2\omega^2A_R^\dagger(\nu)A_R(\nu) + \alpha\omega N_S[A_R^\dagger(\nu), A_R(\nu)]\right).\end{aligned} \tag{9}$$

Adding together the three contributions the commutator $[\mathcal{A}^\dagger(\omega), H_{SR}]$ is reduced to

$$\begin{aligned}[\mathcal{A}^\dagger(\omega), H_{SR}] &= -\omega\mathcal{A}^\dagger(\omega) - g^2A_S^\dagger(\omega)\sum_{\nu\in\mathcal{E}_R}\frac{1}{\nu}\left(\alpha^2\omega^2A_R^\dagger(\nu)A_R(\nu) + \alpha\omega N_S[A_R^\dagger(\nu), A_R(\nu)]\right) + \mathcal{O}\left(\frac{g^3}{|\nu|^3}\right)\\ &= -\omega\mathcal{A}^\dagger(\omega) - g^2\alpha\omega\sum_{\nu\in\mathcal{E}_R}\frac{1}{\nu}\left(N_SA_S^\dagger(\omega)A_R^\dagger(\nu)A_R(\nu) - A_S^\dagger(\omega)N_SA_R(\nu)A_R^\dagger(\nu)\right) + \mathcal{O}\left(\frac{g^3}{|\nu|^3}\right).\end{aligned} \tag{10}$$

Substituting in (3) we obtain,

$$\begin{aligned}(3) = &-\sum_{\omega\in\mathcal{E}_S}\omega G_j(\omega)\langle\mathcal{A}^\dagger(\omega)\mathcal{A}(\omega)\rangle_{\rho_{SR}^I}\\ &-g^2\sum_{\omega\in\mathcal{E}_S,\nu\in\mathcal{E}_R}\Gamma_j(\omega)\frac{\alpha\omega}{\nu}\mathrm{Tr}\left\{\rho_{SR}^I\left(N_SA_S^\dagger(\omega)A_R^\dagger(\nu)A_R(\nu) - A_S^\dagger(\omega)N_SA_R(\nu)A_R^\dagger(\nu)\right)\mathcal{A}(\omega)\right\} + c.c.\\ &+\mathcal{O}\left(\frac{g^3}{|\nu|^3}\right)\end{aligned} \tag{11}$$

The second line of the above expression of the term (3) can be simplified as follows

$$\begin{aligned}&-g^2\sum_{\omega\in\mathcal{E}_S,\nu\in\mathcal{E}_R}\Gamma_j(\omega)\frac{\alpha\omega}{\nu}\mathrm{Tr}\left\{\rho_{SR}^I\left(N_SA_S^\dagger(\omega)A_R^\dagger(\nu)A_R(\nu) - A_S^\dagger(\omega)N_SA_R(\nu)A_R^\dagger(\nu)\right)\mathcal{A}(\omega)\right\} + c.c.\\ &= -g^2\sum_{\omega\in\mathcal{E}_S,\nu\in\mathcal{E}_R}\Gamma_j(\omega)\frac{\alpha\omega}{\nu}\mathrm{Tr}\left\{\rho_{SR}^I\left(N_SA_S^\dagger(\omega)A_R^\dagger(\nu)A_R(\nu) - A_S^\dagger(\omega)N_SA_R(\nu)A_R^\dagger(\nu)\right)A_S(\omega)\right\} + c.c. + \mathcal{O}\left(\frac{g^3}{|\nu|^3}\right)\\ &= -g^2\sum_{\omega\in\mathcal{E}_S,\nu\in\mathcal{E}_R}\Gamma_j(\omega)\frac{\alpha\omega}{\nu}\mathrm{Tr}\left\{\rho_{SR}^I\left(N_SA_S^\dagger(\omega)A_R^\dagger(\nu)A_R(\nu) + A_S^\dagger(\omega)N_SA_R^\dagger(\nu)A_R(\nu)\right)A_S(\omega)\right\} + c.c. + \mathcal{O}\left(\frac{g^3}{|\nu|^3}\right)\\ &= -g^2\sum_{\omega\in\mathcal{E}_S,\nu\in\mathcal{E}_R}\Gamma_j(\omega)\frac{\alpha\omega}{\nu}\mathrm{Tr}\left\{\rho_{SR}^I[N_S, A_S^\dagger(\omega)]_+A_S(\omega)A_R^\dagger(\nu)A_R(\nu)\right\} + c.c. + \mathcal{O}\left(\frac{g^3}{|\nu|^3}\right)\\ &= -g^2\sum_{\omega\in\mathcal{E}_S,\nu\in\mathcal{E}_R}G_j(\omega)\frac{\alpha\omega}{\nu}\mathrm{Tr}\left\{\rho_{SR}^I[N_S, A_S^\dagger(\omega)]_+A_S(\omega)A_R^\dagger(\nu)A_R(\nu)\right\} + \mathcal{O}\left(\frac{g^3}{|\nu|^3}\right),\end{aligned} \tag{12}$$

where $[X,Y]_+ := XY+YX$ denotes the anti-commutator of X and Y. Note that for the bath H the above contribution is trivially null (since $G_H(\omega) = 0$). For the bath C, since the above term is already issued from second-order processes we can approximate ρ_S^I by ρ_S^{eq} (so that $[N_S, \rho_{SR}^I] = \mathcal{O}(g/|\nu|)$) and use (1), and we get

$$\sum_{\omega\in\mathcal{E}_S,\nu\in\mathcal{E}_R}G_C(\omega)\frac{\alpha\omega}{\nu}\mathrm{Tr}\left\{\rho_{SR}^I[N_S, A_S^\dagger(\omega)]_+A_S(\omega)A_R^\dagger(\nu)A_R(\nu)\right\} = \mathcal{O}(g/|\nu|) \tag{13}$$

so that the term (3) is reduced to

$$(3) = -\sum_{\omega\in\mathcal{E}_S} \omega G_C(\omega)\langle \mathcal{A}^\dagger(\omega)\mathcal{A}(\omega)\rangle_{\rho^I_{SR}} + \mathcal{O}\left(\frac{g^3}{|\nu|^3}\right), \tag{14}$$

for the bath C, and $(3) = 0$ for the bath H.

Using the expression of $\mathcal{A}(\omega)$ indicated in Methods, we derive the following expression for $\mathcal{A}^\dagger(\omega)\mathcal{A}(\omega)$,

$$\begin{aligned}
\mathcal{A}^\dagger(\omega)\mathcal{A}(\omega) &= A_S^\dagger(\omega)A_S(\omega) - A_S^\dagger(\omega)A_S(\omega)\sum_{\nu_1,\nu_2\in\mathcal{E}_R}\frac{g^2\alpha^2\omega^2}{\nu_1\nu_2}A_R(\nu_1)A_R(\nu_2) - 2\sum_{\nu\in\mathcal{E}_R}\frac{g^2\alpha^2\omega^2}{\nu^2}A_S^\dagger(\omega)A_S(\omega)A_R^\dagger(\nu)A_R(\nu)\\
&\quad + g^2\sum_{\nu_1\neq-\nu_2}\frac{1}{\nu_2(\nu_1+\nu_2)}\Big(\alpha^2\omega^2A_S^\dagger(\omega)A_S(\omega)A_R(\nu_1)A_R(\nu_2) + \alpha\omega A_S^\dagger(\omega)N_SA_S(\omega)[A_R(\nu_1),A_R(\nu_2)]\Big)\\
&\quad + g^2\sum_{\nu_1\neq-\nu_2}\frac{1}{\nu_2(\nu_1+\nu_2)}\Big(\alpha^2\omega^2A_S^\dagger(\omega)A_S(\omega)A_R(\nu_1)A_R(\nu_2) - \alpha\omega A_S^\dagger(\omega)A_S(\omega)N_S[A_R(\nu_1),A_R(\nu_2)]\Big)\\
&\quad + \mathcal{O}\left(\frac{g^3}{|\nu|^3}\right)\\
&= A_S^\dagger(\omega)A_S(\omega)\Bigg[1 - \sum_{\nu_1,\nu_2\in\mathcal{E}_R}\frac{g^2\alpha^2\omega^2}{\nu_1\nu_2}A_R(\nu_1)A_R(\nu_2) - 2\sum_{\nu\in\mathcal{E}_R}\frac{g^2\alpha^2\omega^2}{\nu^2}A_R^\dagger(\nu)A_R(\nu)\\
&\quad + \sum_{\nu_1\neq-\nu_2}\frac{g^2\alpha^2\omega^2}{\nu_2(\nu_1+\nu_2)}[A_R(\nu_1),A_R(\nu_2)]_+\Bigg] + \mathcal{O}\left(\frac{g^3}{|\nu|^3}\right)\\
&= A_S^\dagger(\omega)A_S(\omega)\Bigg[1 - \sum_{\nu_1,\nu_2\in\mathcal{E}_R}\frac{g^2\alpha^2\omega^2}{\nu_1\nu_2}A_R(\nu_1)A_R(\nu_2) - 2\sum_{\nu\in\mathcal{E}_R}\frac{g^2\alpha^2\omega^2}{\nu^2}A_R^\dagger(\nu)A_R(\nu)\\
&\quad + \sum_{\nu_1\neq-\nu_2}\frac{g^2\alpha^2\omega^2}{\nu_1\nu_2}A_R(\nu_1)A_R(\nu_2)\Bigg] + \mathcal{O}\left(\frac{g^3}{|\nu|^3}\right)\\
&= A_S^\dagger(\omega)A_S(\omega)\left[1 - \sum_{\nu\in\mathcal{E}_R}\frac{g^2\alpha^2\omega^2}{\nu^2}A_R^\dagger(\nu)A_R(\nu)\right] + \mathcal{O}\left(\frac{g^3}{|\nu|^3}\right)
\end{aligned} \tag{15}$$

Finally the term (3) is reduced to

$$\begin{aligned}
(3) &= -\sum_{\omega\in\mathcal{E}_S}\omega G_j(\omega)\left[\langle A_S^\dagger(\omega)A_S(\omega)\rangle_{\rho^I_{SR}} - g^2\alpha^2\omega^2\sum_{\nu\in\mathcal{E}_R}\frac{1}{\nu^2}\langle A_S^\dagger(\omega)A_S(\omega)A_R^\dagger(\nu)A_R(\nu)\rangle_{\rho^I_{SR}}\right] + \mathcal{O}\left(\frac{g^3}{|\nu|^3}\right)\\
&= -\sum_{\omega\in\mathcal{E}_S}\omega G_j(\omega)\langle A_S^\dagger(\omega)A_S(\omega)\rangle_{\rho^I_{SR}} + \mathcal{O}\left(\frac{g^3}{|\nu|^3}\right)
\end{aligned} \tag{16}$$

using the identity (1) in the last line (since the terms are already of second order in g/ν).

2. *Contribution from the term* (4)

The commutator $[\mathcal{A}^\dagger(\omega+\nu), H_{SR}]$ gives

$$[\mathcal{A}^\dagger(\omega+\nu), H_{SR}] = -(\omega+\nu)\mathcal{A}^\dagger(\omega+\nu), \tag{17}$$

so that

$$\begin{aligned}
(4) &= -\sum_{\omega\in\mathcal{E}_S,\nu\in\mathcal{E}_R}(\omega+\nu)\Gamma_j(\omega+\nu)\mathrm{Tr}\{\rho^I_{SR}\mathcal{A}^\dagger(\omega+\nu)\mathcal{A}(\omega+\nu)\} + c.c\\
&= -\sum_{\omega\in\mathcal{E}_S,\nu\in\mathcal{E}_R}(\omega+\nu)G_j(\omega+\nu)\frac{g^2\alpha^2\omega^2}{\nu^2}\langle A_S^\dagger(\omega)A_S(\omega)A_R^\dagger(\nu)A_R(\nu)\rangle_{\rho^I_{SR}}
\end{aligned} \tag{18}$$

3. Contribution from the term (5)

Using the expression of the dissipative map $\Lambda_{j,t}$ given in Methods, we have,

$$
\begin{aligned}
(5) &= \mathrm{Tr}\{\Lambda_{j,t}(\rho_{SR}^I)H_{SR}\} \\
&= -i\sum_{\omega\in\mathcal{E}_S}\partial_\omega\Gamma_j(\omega)\mathrm{Tr}\{\rho_{SR}^I[H_{SR},A_S^\dagger(\omega)]\mathcal{C}(\omega)\}+c.c. \\
&\quad+\sum_{\omega,\omega'\in\mathcal{E}_S}\Gamma_j(\omega)te^{i(\omega'-\omega)t}\mathrm{Tr}\left\{\rho_{SR}^I\Big([A_S^\dagger(\omega'),H_{SR}]\mathcal{C}(\omega)+[\mathcal{C}^\dagger(\omega'),H_{SR}]A_S(\omega)\Big)\right\}+c.c.. \qquad (19)
\end{aligned}
$$

We are again dealing with terms from second-order so that we can use the approximation $\rho_S^I=\rho_S^{eq}+\mathcal{O}(g/|\nu|)$ and the relations $[H_{SR},A_S^\dagger(\omega)]=\omega A_S^\dagger(\omega)+\mathcal{O}(g/|\nu|)$, $[A_S^\dagger(\omega'),H_{SR}]=-\omega' A_S^\dagger(\omega')+\mathcal{O}(g/|\nu|)$ and $[\mathcal{C}^\dagger(\omega'),H_{SR}]=-\omega'\mathcal{C}^\dagger(\omega')+\mathcal{O}(g/|\nu|)$, yielding,

$$
\begin{aligned}
(5) &= -i\sum_{\omega\in\mathcal{E}_S}\omega\partial_\omega\Gamma_j(\omega)\mathrm{Tr}\{\rho_{SR}^IA_S^\dagger(\omega)\mathcal{C}(\omega)\}+\mathcal{O}\left(\frac{g^3}{|\nu|^3}\right)+c.c. \\
&\quad-\sum_{\omega,\omega'\in\mathcal{E}_S}\omega'\Gamma_j(\omega)te^{i(\omega'-\omega)t}\mathrm{Tr}\left\{\rho_{SR}^I\Big(A_S^\dagger(\omega')\mathcal{C}(\omega)+\mathcal{C}^\dagger(\omega')A_S(\omega)\Big)\right\}+\mathcal{O}\left(\frac{g^3}{|\nu|^3}\right)+c.c.. \qquad (20)
\end{aligned}
$$

We now derive a useful identity

$$
\begin{aligned}
\mathrm{Tr}\{\rho_{SR}^I\mathcal{C}^\dagger(\omega')A_S(\omega)\} &= -ig^2\alpha^2\sum_{\nu\in\mathcal{E}_R}\frac{1}{\nu}\langle[A_R(\nu)^\dagger,A_R(\nu)]\rangle_{\rho_R^I}\mathrm{Tr}\{\rho_S^I[A_S^\dagger(\omega'),H_S^2]A_S(\omega)\}+\mathcal{O}\left(\frac{g^3}{|\nu|^3}\right) \\
&= -ig^2\alpha^2\sum_{\nu\in\mathcal{E}_R}\frac{1}{\nu}\langle[A_R(\nu)^\dagger,A_R(\nu)]\rangle_{\rho_R^I}\mathrm{Tr}\{[H_S^2,A_S(\omega)\rho_S^I]A_S^\dagger(\omega')\}+\mathcal{O}\left(\frac{g^3}{|\nu|^3}\right) \\
&= -ig^2\alpha^2\sum_{\nu\in\mathcal{E}_R}\frac{1}{\nu}\langle[A_R(\nu)^\dagger,A_R(\nu)]\rangle_{\rho_R^I}\mathrm{Tr}\{[H_S^2,A_S(\omega)]\rho_S^IA_S^\dagger(\omega')\}+\mathcal{O}\left(\frac{g^3}{|\nu|^3}\right) \\
&= -\mathrm{Tr}\{\rho_{SR}^IA_S^\dagger(\omega')\mathcal{C}(\omega)\}+\mathcal{O}\left(\frac{g^3}{|\nu|^3}\right), \qquad (21)
\end{aligned}
$$

where we again used $\rho_S^I=\rho_S^{eq}+\mathcal{O}(g/|\nu|)$. From the above identity (21) one can simplify the term (5) to

$$
\begin{aligned}
(5) &= -i\sum_{\omega\in\mathcal{E}_S}\omega\partial_\omega G_j(\omega)\mathrm{Tr}\{\rho_{SR}^IA_S^\dagger(\omega)\mathcal{C}(\omega)\}+\mathcal{O}\left(\frac{g^3}{|\nu|^3}\right) \\
&= -i\sum_{\omega\geq0}\omega G_j'(\omega)\mathrm{Tr}\{\rho_{SR}^IA_S^\dagger(\omega)\mathcal{C}(\omega)\}-\omega G_j'(-\omega)\mathrm{Tr}\{\rho_{SR}^IA_S^\dagger(-\omega)\mathcal{C}(-\omega)\}+\mathcal{O}\left(\frac{g^3}{|\nu|^3}\right), \qquad (22)
\end{aligned}
$$

with the notation $G_j'(\omega):=\partial_\omega G_j(\omega)$. We recall that for the bath H the contribution of the term (5) is trivially null ($G_H(\omega)=0$ for all $\omega\in\mathcal{E}_S$). For the bath C the expression of (5) can be simplified further. Using (1) one can show in a similar way

$$
\mathrm{Tr}\{\rho_{SR}^IA_S(\omega)\mathcal{C}^\dagger(\omega)\}=-e^{\omega/T_C}\mathrm{Tr}\{\rho_{SR}^IA_S^\dagger(\omega)\mathcal{C}(\omega)\}. \qquad (23)
$$

Furthermore, the equality

$$
G_j'(-\omega)=e^{-\omega/T_j}[G_j(\omega)/T_j-G_j'(\omega)] \qquad (24)
$$

holds for thermal baths. Combining the two last equalities (23) and (24) we finally obtain,

$$
\begin{aligned}
(5) &= -i\sum_{\omega\geq0}\omega\langle A_S^\dagger(\omega)\mathcal{C}(\omega)\rangle_{\rho_{SR}^I}[G_C'(\omega)+e^{\omega/T_C}G_C'(-\omega)]+\mathcal{O}\left(\frac{g^3}{|\nu|^3}\right) \\
&= -i\sum_{\omega\geq0}\frac{\omega}{T_C}\langle A_S^\dagger(\omega)\mathcal{C}(\omega)\rangle_{\rho_{SR}^I}G_C(\omega)+\mathcal{O}\left(\frac{g^3}{|\nu|^3}\right).
\end{aligned}
$$

(25)

From the expression of $\mathcal{C}(\omega)$ (in Methods) and using the identity (1) one can rewrite (5) in the form

$$(5) = -\sum_{\omega\in\mathcal{E}_S,\nu\in\mathcal{E}_R} 2\nu T_j^{-1}\frac{\alpha^2\omega^2 g^2}{\nu^2} G_j(\omega)\langle A_S^\dagger(\omega)A_S(\omega)H_S A_R^\dagger(\nu)A_R(\nu)\rangle_{\rho_{SR}^I} + \mathcal{O}\left(\frac{g^3}{|\nu|^3}\right), \tag{26}$$

which also gives the right expression for H, namely $(5) = 0$.

4. *Final expression for $\dot{Q}_{SR/j}$*

Combining terms (3), (4), and (5) we obtain finally, valid for $j = H, C$,

$$\begin{aligned}\dot{Q}_{SR/j} = &-\sum_{\omega\in\mathcal{E}_S}\omega G_j(\omega)\langle A_S^\dagger(\omega)A_S(\omega)\rangle_{\rho_S^I}\\ &-\sum_{\omega\in\mathcal{E}_S,\nu\in\mathcal{E}_R}(\omega+\nu)G_j(\omega+\nu)\frac{g^2\alpha^2\omega^2}{\nu^2}\langle A_S^\dagger(\omega)A_S(\omega)A_R^\dagger(\nu)A_R(\nu)\rangle_{\rho_{SR}^I}\\ &-\sum_{\omega\in\mathcal{E}_S,\nu\in\mathcal{E}_R}2\nu T_j^{-1}\frac{\alpha^2\omega^2 g^2}{\nu^2}G_j(\omega)\langle A_S^\dagger(\omega)A_S(\omega)H_S A_R^\dagger(\nu)A_R(\nu)\rangle_{\rho_{SR}^I} + \mathcal{O}\left(\frac{g^3}{|\nu|^3}\right).\end{aligned} \tag{27}$$

Since we neglect contributions of order higher than $g^2/|\nu|^2$ one can once again approximate ρ_S^I by ρ_S^{eq} (or ρ_{SR}^{eq} by $\rho_S^{eq}\rho_R^I$), yielding,

$$\langle A_S^\dagger(\omega)A_S(\omega)A_R^\dagger(\nu)A_R(\nu)\rangle_{\rho_{SR}^I} = \langle A_S^\dagger(\omega)A_S(\omega)\rangle_{\rho_S^{eq}}\langle A_R^\dagger(\nu)A_R(\nu)\rangle_{\rho_R^I} + \mathcal{O}\left(\frac{g}{|\nu|}\right), \tag{28}$$

and,

$$\langle A_S^\dagger(\omega)A_S(\omega)H_S A_R^\dagger(\nu)A_R(\nu)\rangle_{\rho_{SR}^I} = \langle A_S^\dagger(\omega)A_S(\omega)H_S\rangle_{\rho_S^{eq}}\langle A_R^\dagger(\nu)A_R(\nu)\rangle_{\rho_R^I} + \mathcal{O}\left(\frac{g}{|\nu|}\right). \tag{29}$$

However, the first line of (27) is of order 0 in $g/|\nu|$ so that we cannot approximate $\langle A_S^\dagger(\omega)A_S(\omega)\rangle_{\rho_S^I}$ by $\langle A_S^\dagger(\omega)A_S(\omega)\rangle_{\rho_S^{eq}}$. A derivation of an expression of $\langle A_S^\dagger(\omega)A_S(\omega)\rangle_{\rho_S^I}$ to second order in $g/|\nu|$ is presented in Section II.

B. Expression of $\dot{Q}_{S/j}$

From the above calculations we can easily derive the following expression for $\dot{Q}_{S/j}$, the heat flow entering S only,

$$\begin{aligned}\dot{Q}_{S/j} = &-\sum_{\omega\in\mathcal{E}_S}\omega G_j(\omega)\langle A_S^\dagger(\omega)A_S(\omega)\rangle_{\rho_S^I}\\ &-\sum_{\omega\in\mathcal{E}_S,\nu\in\mathcal{E}_R}\omega G_j(\omega+\nu)\frac{g^2\alpha^2\omega^2}{\nu^2}\langle A_S^\dagger(\omega)A_S(\omega)\rangle_{\rho_S^{eq}}\langle A_R^\dagger(\nu)A_R(\nu)\rangle_{\rho_R^I}\\ &-\sum_{\omega\in\mathcal{E}_S,\nu\in\mathcal{E}_R}2\nu T_j^{-1}\frac{\alpha^2\omega^2 g^2}{\nu^2}G_j(\omega)\langle A_S^\dagger(\omega)A_S(\omega)H_S\rangle_{\rho_S^{eq}}\langle A_R^\dagger(\nu)A_R(\nu)\rangle_{\rho_R^I} + \mathcal{O}\left(\frac{g^3}{|\nu|^3}\right).\end{aligned} \tag{30}$$

The unique difference from the expression of $\dot{Q}_{SR/j}$ comes from the second line which carries an energy ω instead of $\omega+\nu$.

II. EVALUATION OF $\langle A_S^\dagger(\omega)A_S(\omega)\rangle_{\rho_S^I}$ TO 2^{d} ORDER IN $g/|\nu|$

In Section I we obtained expressions of the different heat flows up to order g^2/ν^2. Accordingly, the expectation value $\langle A_S^\dagger(\omega)A_S(\omega)\rangle_{\rho_S^I}$ therein has to be evaluated to the same order g^2/ν^2. To do so we take the time derivative of

$\langle A_S^\dagger(\omega)A_S(\omega)\rangle_{\rho_S^I}$ and keep only terms up to second order in $g/|\nu|$. The obtained differential equation can be easily solved when S is a harmonic oscillator or a two-level system. In the remainder of the Supplemental Material we focus on such situations and denote by ω_0 the transition frequency of S, implying $\mathcal{E}_S = \{-\omega_0, \omega_0\}$. To simplify further the model and the results we assume that C is resonant only with S, and that H is resonant with only one transition energy of SR, denoted by $\omega_0+\nu_0$ with $\nu_0 \geq 0$. Then, $G_H(\omega_0+\nu) = 0$ for all $\nu \neq \nu_0$. This corresponds to the model considered in the main text. Starting with

$$\frac{d}{dt}\langle A_S^\dagger(\omega)A_S(\omega)\rangle_{\rho_S^I} = \mathrm{Tr}\{\dot\rho_S^I A_S^\dagger(\omega)A_S(\omega)\} = \mathrm{Tr}\{\dot\rho_{SR}^I A_S^\dagger(\omega)A_S(\omega)\}, \tag{31}$$

we inject expression from Methods of $\dot\rho_{SR}^I$ in the above one and after similar calculations as in the last section I we obtain

$$\begin{aligned}\frac{d}{dt}\langle A_S^\dagger(\bar\omega)A_S(\bar\omega)\rangle_{\rho_S^I} = &\sum_{\omega=\pm\omega_0} G_C(\omega)\langle [A_S^\dagger(\omega), A_S^\dagger(\bar\omega)A_S(\bar\omega)]A_S(\omega)\rangle_{\rho_S^I}\\ &+\sum_{\omega+\nu=\pm(\omega_0+\nu_0)} G_H(\omega+\nu)\frac{g^2\alpha^2\omega^2}{\nu^2}\langle A_R^\dagger(\nu)A_R(\nu)\rangle_{\rho_R^I}\langle [A_S^\dagger(\omega), A_S^\dagger(\bar\omega)A_S(\bar\omega)]A_S(\omega)\rangle_{\rho_S^I}\\ &+\sum_{\omega=\pm\omega_0,\nu\in\mathcal{E}_R} G_C(\omega)g^2\alpha^2\frac{\omega}{\nu}T_C^{-1}\langle [A_R^\dagger(\nu), A_R(\nu)]\rangle_{\rho_R^I}\langle [A_S^\dagger(\omega), A_S^\dagger(\bar\omega)A_S(\bar\omega)]A_S(\omega)H_S\rangle_{\rho_S^I}\\ &+\mathcal{O}\left(\frac{g^3}{|\nu|^3}\right).\end{aligned} \tag{32}$$

To continue we need to compute the commutators appearing in the differential equation. We do so in the following Sections when S is a harmonic oscillator (Section II A) and a two-level system (Section II B).

A. Harmonic oscillators

We assume here that S is a harmonic oscillator of frequency ω_0 and a, $a^\dagger$ are the annihilation and creation operators. Harmonic oscillators usually couple to baths through quadratures operators. We therefore choose P_S of the following form,

$$P_S = ca^\dagger + c^* a, \tag{33}$$

where c is a complex number. As a consequence, $A_S(\omega_0) = c^* a$ and $A_S(-\omega_0) = ca^\dagger$. Equation (32) is then simplified to

$$\frac{d}{dt}\langle a^\dagger a\rangle_{\rho_S^I} = -\lambda\langle a^\dagger a\rangle_{\rho_S^I} + r, \tag{34}$$

where

$$\lambda = |c|^4\left\{G_C(\omega_0) - G_C(-\omega_0) + \frac{g^2\alpha^2\omega_0^2}{\nu_0^2}\left[G_H(\omega_0+\nu_0)\langle A_R^\dagger(\nu_0)A_R(\nu_0)\rangle_{\rho_R^I} - G_H(-\omega_0-\nu_0)\langle A_R(\nu_0)A_R^\dagger(\nu_0)\rangle_{\rho_R^I}\right]\right\}, \tag{35}$$

and

$$\begin{aligned}r = |c|^4 G_C(-\omega_0) + |c|^4 g^2\alpha^2\omega_0^2\Bigg\{&\frac{G_H(-\omega_0-\nu_0)}{\nu_0^2}\langle A_R(\nu_0)A_R^\dagger(\nu_0)\rangle_{\rho_R^I} - \left[G_C(\omega_0)\langle (a^\dagger a)^2\rangle_{\rho_S^I} + G_C(-\omega_0)\langle aa^{\dagger 2}a\rangle_{\rho_S^I}\right]\\ &\times\sum_{\nu\in\mathcal{E}_R}\frac{2T_C^{-1}}{\nu}\langle A_R^\dagger(\nu)A_R(\nu)\rangle_{\rho_R^I}\Bigg\} + \mathcal{O}(g^3/|\nu|^3).\end{aligned} \tag{36}$$

In the following we set $c = 1$ since the phase of c has no observable influence (and the amplitude of c can be included in $G(\omega)$).

A quick analysis of the dynamics of $\langle A_R(\nu)A_R^\dagger(\nu)\rangle_{\rho_R^I}$ reveals that its time derivative is of order 2 in $g/|\nu|$ (the contribution of $\mathcal{L}_0$ clearly gives zero whereas the contribution of $g\mathcal{L}_1$ turns out to be of order g^2/ν^2 when $t \gg \tau_1 := G_C^{-1}(\omega_0)$. One obtains from this observation that λ varies very slowly, at a rate of order g^4/ν^4, so that it can safely be

taken as constant. The same conclusion can be drawn for r. Then we conclude that a good approximation of $\langle a^\dagger a\rangle_{\rho_S^I}$ is

$$\langle a^\dagger a\rangle_{\rho_S^I} = e^{-\lambda t}\langle a^\dagger a\rangle_{\rho_S(0)} + \frac{1-e^{-\lambda t}}{\lambda} r =_{t\gg\tau_1} \frac{r}{\lambda}. \tag{37}$$

Substituting in (27), (30), (51) and using (1) one obtains for the heat flows

$$\dot{Q}_{SR/C} = \omega_0 \frac{g^2\alpha^2\omega_0^2}{\nu_0^2}\frac{G_C(\omega_0)G_H(\omega_0+\nu_0)}{G_C(\omega_0)-G_C(-\omega_0)}\left(e^{-\frac{\omega_0}{T_C}}\langle A_R^\dagger(\nu_0)A_R(\nu_0)\rangle_{\rho_R^I} - e^{-\frac{\omega_0+\nu_0}{T_H}}\langle A_R(\nu_0)A_R^\dagger(\nu_0)\rangle_{\rho_R^I}\right) + \mathcal{O}(g^3/\nu_0^3), \tag{38}$$

$$\begin{aligned}\dot{Q}_{SR/H} &= -(\omega_0+\nu_0)\frac{g^2\alpha^2\omega_0^2}{\nu_0^2}\frac{G_C(\omega_0)G_H(\omega_0+\nu_0)}{G_C(\omega_0)-G_C(-\omega_0)}\left(e^{-\frac{\omega_0}{T_C}}\langle A_R^\dagger(\nu_0)A_R(\nu_0)\rangle_{\rho_R^I} - e^{-\frac{\omega_0+\nu_0}{T_H}}\langle A_R(\nu_0)A_R^\dagger(\nu_0)\rangle_{\rho_R^I}\right) + \mathcal{O}(g^3/\nu_0^3)\\ &= -\frac{\omega_0+\nu_0}{\omega_0}\dot{Q}_{SR/C} + \mathcal{O}\left(\frac{g^3}{\nu_0^3}\right),\end{aligned} \tag{39}$$

$$\dot{Q}_{S/C} = -\dot{Q}_{S/H} + \mathcal{O}\left(\frac{g^3}{\nu^3}\right) = \dot{Q}_{SR/C} + \mathcal{O}\left(\frac{g^3}{\nu^3}\right). \tag{40}$$

Equation (40) implies in particular that $\dot{E}_S = \mathcal{O}\left(\frac{g^3}{\nu_0^3}\right)$. This is the usual condition of steady state for continuous thermal machines [1–4] mentioned in the main text and valid for times t much bigger than $\tau_{es} = [G_C(\omega_0)]^{-1}$.

B. Two-level systems

We assume is this section that S is a two-level system of transition frequency ω_0. Such system usually couples to baths through the operators σ_+ and σ_- (the Pauli matrices) so that a natural choice for P_S is

$$P_S = c\sigma_+ + c^*\sigma_-. \tag{41}$$

where c is a complex number. As a consequence $A_S(\omega_0) = c^*\sigma_-$, $A_S(-\omega_0) = c\sigma_+$, $\langle A_S(\omega_0)A_S^\dagger(\omega_0)\rangle_{\rho_S^I} = |c|^2\rho_{gg}$, and $\langle A_S^\dagger(\omega_0)A_S(\omega_0)\rangle_{\rho_S^I} = |c|^2\rho_{ee}$, where $\rho_{gg} := \langle g|\rho_S^I|g\rangle$ and $\rho_{ee} := \langle e|\rho_S^I|e\rangle$, being $|g\rangle$ and $|e\rangle$ the ground and excited state of S respectively.
From (32) we obtain the following dynamics

$$\dot{\rho}_{ee} = -\dot{\rho}_{gg} = -R_+\rho_{ee} + R_-\rho_{gg}, \tag{42}$$

with

$$\begin{aligned}R_\pm &= |c|^4 G_C(\pm\omega_0) + |c|^4 g^2\alpha^2\omega_0^2\left[\frac{G_H(\pm(\omega_0+\nu_0))}{\nu_0^2}\langle A_R^\dagger(\pm\nu_0)A_R(\pm\nu_0)\rangle_{\rho_R^I} + G_C(\pm\omega_0)\sum_{\nu\in\mathcal{E}_R}\frac{T_C^{-1}}{\nu}\langle A_R^\dagger(\nu)A_R(\nu)\rangle_{\rho_R^I}\right]\\ &\quad + \mathcal{O}\left(\frac{g^3}{|\nu|^3}\right).\end{aligned} \tag{43}$$

As in the previous Section the analysis of the dynamics of $\langle A_R^\dagger(\nu)A_R(\nu)\rangle_{\rho_R^I}$ reveals that its time derivative is of order g^2/ν^2 implying that $\dot{R}_\pm = \mathcal{O}(g^4/\nu^4)$, justifying that we can safely take $R_\pm$ as constant. The dynamics of $\rho_{ee} = 1-\rho_{gg}$ is then

$$\rho_{ee} = e^{-Rt}\rho_{ee}(0) + \frac{1-e^{-Rt}}{R}R_- =_{t\gg\tau_1} \frac{R_-}{R}, \tag{44}$$

where $R := R_+ + R_-$. Substituting in (27), (30), (51) and using (1) one obtains for the heat flows similar expressions as in the previous Section II A,

$$\dot{Q}_{SR/C} = \omega_0 \frac{g^2\alpha^2\omega_0^2}{\nu_0^2}\frac{G_C(\omega_0)G_H(\omega_0+\nu_0)}{G_C(\omega_0)+G_C(-\omega_0)}\left(e^{-\frac{\omega_0}{T_C}}\langle A_R^\dagger(\nu_0)A_R(\nu_0)\rangle_{\rho_R^I} - e^{-\frac{\omega_0+\nu_0}{T_H}}\langle A_R(\nu_0)A_R^\dagger(\nu_0)\rangle_{\rho_R^I}\right) + \mathcal{O}(g^3/\nu_0^3), \tag{45}$$

$$\dot{Q}_{SR/H} = -(\omega_0+\nu_0)\frac{g^2\alpha^2\omega_0^2}{\nu_0^2}\frac{G_C(\omega_0)G_H(\omega_0+\nu_0)}{G_C(\omega_0)+G_C(-\omega_0)}\left(e^{-\frac{\omega_0}{T_C}}\langle A_R^\dagger(\nu_0)A_R(\nu_0)\rangle_{\rho_R^I} - e^{-\frac{\omega_0+\nu_0}{T_H}}\langle A_R(\nu_0)A_R^\dagger(\nu_0)\rangle_{\rho_R^I}\right) + \mathcal{O}(g^3/\nu_0^3)$$
$$= -\frac{\omega_0+\nu_0}{\omega_0}\dot{Q}_{SR/C} + \mathcal{O}\left(\frac{g^3}{\nu_0^3}\right), \tag{46}$$

$$\dot{Q}_{S/C} = -\dot{Q}_{S/H} + \mathcal{O}\left(\frac{g^3}{\nu^3}\right) = \dot{Q}_{SR/C} + \mathcal{O}\left(\frac{g^3}{\nu^3}\right). \tag{47}$$

Same identities as for harmonic oscillators (but the expression of the heat flows differs slightly, $G_C(\omega_0) + G_C(-\omega_0)$ in the denominator instead of $G_C(\omega_0) - G_C(-\omega_0)$). As in the previous Section, Eq. (47) implies in particular that $\dot{E}_S = \mathcal{O}\left(\frac{g^3}{\nu_0^3}\right)$. This is the usual condition of steady state for continuous thermal machines [1–4] mentioned in the main text and valid for times t much bigger than $\tau_{es} = [G_C(\omega_0)]^{-1}$.

III. EXPRESSION OF $\dot{E}_R$

The internal energy of R is defined as (see also main text) $E_R := \langle H_R\rangle_{\rho_{SR}}$. In term of ρ_{SR}^I, the density matrix of SR in the interaction picture with respect to H_{SR}, the internal energy of R can be re-written as

$$E_R = \langle H_R^I(t)\rangle_{\rho_{SR}^I(t)}, \tag{48}$$

with $H_R^I(t) := e^{itH_{SR}}H_R e^{-itH_{SR}} \neq H_R$. Then, the time derivative of E_R is a sum of two contributions:

$$\dot{E}_R = \mathrm{Tr}\dot{\rho}_{SR}^I(t)H_R^I(t) + \mathrm{Tr}\rho_{SR}^I(t)\dot{H}_R^I(t). \tag{49}$$

Using Eq. (23) of Methods 'Why dispersive coupling?' (with $H_R^I(t)$ instead of H_{SR}) one can show straightforwardly that $\mathrm{Tr}\dot{\rho}_{SR}^I(t)H_R^I(t) = 0$. This is because in the interaction picture with respect to H_{SR}, R does not interact with S and therefore "does not see" the baths. Then, only the second term contribute to $\dot{R}_R$. Up to second order in $g/|\nu|$ one can show that

$$\mathrm{Tr}\rho_{SR}^I(t)\dot{H}_R^I(t) = ig\sum_{\nu\in\mathcal{E}_R}\nu e^{-i\nu t}\langle N_S A_R(\nu)\rangle_{\rho_{SR}^I} + ig^2\sum_{\nu,\nu'\in\mathcal{E}_R}\frac{\nu}{\nu'}\langle N_S^2[A_R(\nu),A_R(\nu')]\rangle_{\rho_{SR}^I}e^{-i\nu t}(e^{-i\nu' t}-1)$$
$$+\mathcal{O}\left(\frac{g^3}{|\nu|^3}\right). \tag{50}$$

The term of second order in g/ν is rapidly oscillating with $\nu \neq \nu'$, resulting in a contribution of higher order (after time-graining), and terms of $\nu' = -\nu$ sum up to zero since $\sum_{\nu\in\mathcal{E}_R}[A_R(\nu), A_R^\dagger(\nu)] = 0$. The first term contains the rapidly oscillating phase $e^{-i\nu t}$ so that the expression of $\langle N_S A_R(\nu)\rangle_{\rho_{SR}^I}$ has to be derived retaining only terms oscillating at the frequency ν, becoming non-oscillating terms after multiplying by the phase $e^{-i\nu t}$. This can be done (for S harmonic oscillator or two-level system) using Eq. (21) of Methods "Why dispersive coupling?" (since Eq. (36) of Methods "Expression of the baths dissipative operators" was obtained by neglecting fast oscillating terms). We finally find

$$\dot{E}_R = -\sum_{\omega\in\mathcal{E}_S,\nu\in\mathcal{E}_R}\nu G_H(\omega+\nu)\frac{g^2\alpha^2\omega^2}{\nu^2}\langle A_S^\dagger(\omega)A_S(\omega)\rangle_{\rho_S^{eq}}\langle A_R^\dagger(\nu)A_R(\nu)\rangle_{\rho_R^I} + \mathcal{O}\left(\frac{g^3}{|\nu|^3}\right). \tag{51}$$

Comparing with Eqs. (39) and (46) when S is a harmonic oscillator and a two-level system, respectively, one obtains the following fundamental relation, valid for $t \gg \tau_{es}$,

$$\dot{E}_R = \frac{\nu_0}{\omega_0+\nu_0}\dot{Q}_{SR/H} + \mathcal{O}\left(\frac{g^3}{\nu_0^3}\right) = -\frac{\nu_0}{\omega_0}\dot{Q}_{SR/C} + \mathcal{O}\left(\frac{g^3}{\nu_0^3}\right). \tag{52}$$

IV. REFRIGERATION CONDITIONS AND EFFICIENCY

In Section II we saw that the heat flows $\dot{Q}_{SR/j}$ takes almost the same expression for both two-level systems and harmonic oscillators. Thanks to that the following considerations are valid for both systems. From Section II we have

$$\begin{aligned}\dot{Q}_{SR/C} &= -\frac{\omega_0}{\omega_0+\nu_0}\dot{Q}_{SR/H} \\ &= \omega_0 \frac{g^2\alpha^2\omega_0^2}{\nu_0^2}\frac{G_C(\omega_0)G_H(\omega_0+\nu_0)}{G_C(\omega_0)\pm G_C(-\omega_0)}\left(e^{-\frac{\omega_0}{T_C}}\langle A_R^\dagger(\nu_0)A_R(\nu_0)\rangle_{\rho_R^I} - e^{-\frac{\omega_0+\nu_0}{T_H}}\langle A_R(\nu_0)A_R^\dagger(\nu_0)\rangle_{\rho_R^I}\right) + \mathcal{O}\left(\frac{g^3}{\nu_0^3}\right).\end{aligned} \tag{53}$$

The $\pm$ at the denominator corresponds to the possibility of S being a harmonic oscillator or a two-level system. The refrigeration condition corresponds to $\dot{Q}_{SR/C} \geq 0$, which implies from (52) $\dot{E}_R \leq 0$, meaning that R supplies energy to the refrigerator. The refrigeration condition is

$$\frac{\omega_0+\nu_0}{T_H} - \frac{\omega_0}{T_C} \geq \ln\frac{\langle A_R(\nu_0)A_R^\dagger(\nu_0)\rangle_{\rho_R^I}}{\langle A_R^\dagger(\nu_0)A_R(\nu_0)\rangle_{\rho_R^I}}. \tag{54}$$

We defined the apparent temperature of R as

$$\mathcal{T}_R := \nu_0\left(\ln\frac{\langle A_R(\nu_0)A_R^\dagger(\nu_0)\rangle_{\rho_R^I}}{\langle A_R^\dagger(\nu_0)A_R(\nu_0)\rangle_{\rho_R^I}}\right)^{-1}. \tag{55}$$

From (1) (adapted to R) one can show that $\mathcal{T}_R = T_R$ when R is in a thermal state at temperature T_R. More properties of the apparent temperature are mentioned in the main text. The refrigeration condition can be rewritten as

$$\omega_0 \leq \nu_0\frac{T_C}{T_H-T_C}\left(1-\frac{T_H}{\mathcal{T}_R}\right), \tag{56}$$

which is the result announced and discussed in the main text.

The efficiency η is defined as the ratio of the energy extracted from C, $\dot{Q}_{SR/C}$, by the energy invested by R, $-\dot{E}_R$, $\eta := \frac{\dot{Q}_{SR/C}}{-\dot{E}_R}$. From the expressions (52) we have

$$\eta = \frac{\omega_0}{\nu_0} + \mathcal{O}\left(\frac{g^3}{\nu_0^3}\right) \leq \frac{T_C}{T_H-T_C}\left(1-\frac{T_H}{\mathcal{T}_R}\right), \tag{57}$$

and the upper bound is a direct consequence of the above refrigeration condition. This result is also announced and discussed in the main text.

Note that we defined the efficiency in terms of *rates* of the energy flows. Traditionally it is defined in terms of time-integrated flows of energy, i.e. finite difference of energy (between a time t and the initial time). One can show that if we ignore the small time interval of order $\tau_1 = G_C^{-1}(\omega_0)$ before S reaches the steady state, the efficiency $\eta_{int} := \frac{Q_{SR/C}}{-\Delta E_R}$ is also equal to $\frac{\omega_0}{\nu_0} + \mathcal{O}\left(\frac{g^3}{\nu_0^3}\right)$.

V. ENERGY EXTRACTION CONDITIONS AND EFFICIENCY

We derive in this Section the conditions and the efficiency for the reverse operation of refrigeration: the storage in R of energy extracted from the baths. Such storage operation is obtained by reversing the sign of all heat flows with respect to the refrigeration regime. The storage condition is $\dot{E}_R \geq 0$ which from (52) implies $\dot{Q}_{SR/H} \geq 0$ and $\dot{Q}_{SR/C} \leq 0$. From Section II we have

$$\dot{E}_R = -\nu_0\frac{g^2\alpha^2\omega_0^2}{\nu_0^2}\frac{G_C(\omega_0)G_H(\omega_0+\nu_0)}{G_C(\omega_0)+G_C(-\omega_0)}\left(e^{-\frac{\omega_0}{T_C}}\langle A_R^\dagger(\nu_0)A_R(\nu_0)\rangle_{\rho_R^I} - e^{-\frac{\omega_0+\nu_0}{T_H}}\langle A_R(\nu_0)A_R^\dagger(\nu_0)\rangle_{\rho_R^I}\right) + \mathcal{O}(g^3/\nu_0^3), \tag{58}$$

which imposes for the extraction condition,

$$\omega_0 \geq \nu_0 \frac{T_C}{T_H - T_C}\left(1 - \frac{T_H}{\mathcal{T}_R}\right), \tag{59}$$

just the opposite of the refrigeration condition. The efficiency η_e is defined as the energy stored in R, accounted by $\dot{E}_R$, divided by the cost in thermal energy from the hot bath, accounted by $\dot{Q}_{SR/H}$, $\eta_e := \frac{\dot{E}_R}{\dot{Q}_{SR/H}}$. From (52) we have that $\eta_e = \frac{\nu_0}{\omega_0+\nu_0} + \mathcal{O}(g^3/\nu_0^3)$. The storage extraction condition provides the upper bound,

$$\eta_e \leq \left(1 - \frac{T_C}{T_H}\right)\frac{\mathcal{T}_R}{\mathcal{T}_R - T_C}, \tag{60}$$

assuming $\mathcal{T}_R \geq T_C$ (otherwise one can heat up R trivially by thermal contact with C or H). This is the expression mentioned and briefly discussed in the main text.

VI. APPARENT TEMPERATURE OF NON-DEGENERATED SYSTEMS

A. Apparent temperature of squeezed states

In this Section we consider that R is a harmonic oscillator with the usual annihilation operator $A_R(\nu_0) = a$, creation operator $A_R^\dagger(\nu_0) = a^\dagger$, and the free Hamiltonian $H_R = \nu_0 a^\dagger a$, it is straight forward to show that $\mathcal{T}_R := \nu_0\left[\ln\left(1 + \nu_0/E_R\right)\right]^{-1}$, which depends only on the average internal energy E_R. Some consequences of such property are detailed in the main text. As a special case, we consider a squeezed thermal state of squeezing factor r and thermal excitation corresponding to a temperature T_R. The mean energy of such squeezed state is [5]

$$E_R/\nu_0 = \sinh^2 r + (\sinh^2 r + \cosh^2 r)\left(e^{\nu_0/T_R} - 1\right)^{-1}. \tag{61}$$

The expression of the apparent temperature can be rewritten as

$$\mathcal{T}_R = \nu_0\left[\ln\frac{\tanh^2 r + e^{\nu_0/T_R}}{\tanh^2 r e^{\nu_0/T_R} + 1}\right]^{-1}, \tag{62}$$

which is the analogue of the expression used in [6, 7] for the effective temperature characterising the upper bound efficiency in presence of squeezed baths.

B. Apparent temperature of a non-degenerated finite-level system

Let's consider now a N-equidistant-level system. The ladder operator takes the form

$$A_R(\nu_0) = \sum_{n=1}^{N-1} c_{n,n+1}|n\rangle\langle n+1|, \tag{63}$$

where $|n\rangle$ is the eigenstate of the level n and $c_{n,n+1} := \langle n|A_R|n+1\rangle$. The products of the upwards and downwards ladder operators give

$$A_R^\dagger(\nu_0)A_R(\nu_0) = \sum_{n=1}^{N-1} |c_{n,n+1}|^2|n+1\rangle\langle n+1|, \tag{64}$$

$$A_R(\nu_0)A_R^\dagger(\nu_0) = \sum_{n=1}^{N-1} |c_{n,n+1}|^2|n\rangle\langle n|. \tag{65}$$

From the definition of the apparent temperature [8] provided in the main text, we obtain

$$\mathcal{T}_R = \nu_0\left(\log\frac{\sum_{n=1}^{N-1}|c_{n,n+1}|^2\rho_n}{\sum_{n=1}^{N-1}|c_{n,n+1}|^2\rho_{n+1}}\right), \tag{66}$$

where $\rho_n := \langle n|\rho_R^I|n\rangle$ is the population of the level n. For $N \geq 3$ it appears that changing the value of the some ρ_n can alter $\mathcal{T}_R$ but not necessarily the internal energy E_R, and reciprocally. It is more apparent if one assumes that the transition amplitudes $\langle n|A_R|n+1\rangle$ are independent of n implying the following simple expression for the apparent temperature,

$$\mathcal{T}_R = \nu_0 \left(\ln \frac{1-\rho_N}{1-\rho_1} \right)^{-1}. \tag{67}$$

Now we ask the question, given a fixed average energy E_R, what is the maximal achievable value of $\mathcal{T}_R$? A simple way to derive the solution is by answering the reverse question: we look for the smallest E_R compatible with a fixed apparent temperature $\mathcal{T}_R$.

We first assume that $\mathcal{T}_R$ is positive. This fixed the value of ρ_N in term of ρ_1, $\rho_N = 1 - e^{\nu_0/\mathcal{T}_R}(1-\rho_1)$, with ρ_1 restricted to the range of values $1 - e^{\nu_0/\mathcal{T}_R} \leq \rho_1 \leq 1$ in order to ρ_N be in the interval $[0;1]$. In case the population ρ_1 and ρ_N do not sum up to 1, $\rho_1 + \rho_N < 1$, some other levels have to be populated. The choice which leads certainly to the lowest mean energy is populating the lowest available level, the level 2 (assuming $N \geq 3$). Then, we choose $\rho_2 = 1 - \rho_1 - \rho_N$, with the allowed range of values for ρ_1:

$$\frac{e^{\nu_0/\mathcal{T}_R} - 1}{e^{\nu_0/\mathcal{T}_R} + 1} \leq \rho_1 \leq \frac{e^{\nu_0/\mathcal{T}_R}}{e^{\nu_0/\mathcal{T}_R} + 1}. \tag{68}$$

It follows that the double constraint $\rho_N, \rho_2 \in [0;1]$ implies $1 - e^{-\nu_0/\mathcal{T}_R} \leq \rho_1 \leq \frac{e^{\nu_0/\mathcal{T}_R}}{e^{\nu_0/\mathcal{T}_R}+1}$. The mean energy of such a state is $E_R = \nu_0\rho_2 + \nu_0(N-1)\rho_N$, choosing a ground state energy equal to zero. Substituting ρ_2 and ρ_N by their expression in term of ρ_1 we have

$$E_R = \nu_0\{N - 2 + (\rho_1 - 1)[(N-2)e^{\nu_0/\mathcal{T}_R} - 1]\}. \tag{69}$$

For $N \geq 3$, the minimal energy is $E_{R,min}(\mathcal{T}_R) = \nu_0 e^{-\nu_0/\mathcal{T}_R}$, achieved for the minimal allowed value $\rho_1 = 1 - e^{-\nu_0/\mathcal{T}_R}$. Reversing the relation we obtained the maximal apparent temperature for a given energy $E_R \leq \nu_0$,

$$\mathcal{T}_{R,max} = \nu_0[\ln \nu_0/E_R]^{-1}. \tag{70}$$

For $E_R \geq \nu_0$, the maximal apparent temperature is *negative*. Its expression can be derived in the same way as done above but assuming $\mathcal{T}_R$ negative.

Finally, a thermal state at temperature T_R has an internal energy equal to

$$E_R^{th}(T_R) = \nu_0[(e^{\nu_0/T_R} - 1)^{-1} - N(e^{N\nu_0/T_R} - 1)^{-1}]. \tag{71}$$

Then, thermal state of infinite temperature has an energy equal to $\nu_0(N-1)/2$. It follows that for $N \geq 4$, thermal states of *positive* temperatures can be manipulated with no energy change into non-thermal states of *negative* $\mathcal{T}_R$. Substituting E_R^{th} into (70) one can find numerically the maximum of $\mathcal{T}_{R,max}/T_R$.For instance, for $N = 3$, we find $\mathcal{T}_{R,max}/T_R \to 1.5$ (achieved for T_R going to infinity). For $N \geq 4$ the ratio $\mathcal{T}_{R,max}/T_R$ is indeed unbounded since a thermal energy equal to ν_0 (corresponding to a finite positive temperature T_R) is enough to reach an infinite apparent temperature.

[1] A. Levy and R. Kosloff, Phys. Rev. Lett. **108**, 070604 (2012).
[2] R. Kosloff, Entropy **15**, 2100-2128 (2013).
[3] R. Kosloff and A. Levy, Annu. Rev. Phys. Chem. **65**:365-93 (2014).
[4] D. Gelbwaser-Klimovsky and G. Kurizki, Phys. Rev. E **90**, 022102 (2014).
[5] A. Ferraro, S. Olivares, M. Paris, *Gaussian States in Quantum Information*, (Bibliopolis, Napoli, 2005).
[6] Huang, X. L., Wang, T. and Yi, X. X., Phys. Rev. E **86**, 051105 (2012).
[7] L. A. Correa, J. P. Palao, D. Alonso, and G. Adesso, Scientific Reports **4**, 3949 (2014).
[8] C. L. Latune, I. Sinayskiy, and F. Petruccione, arXiv:1803.11495.
